# Effects of HIV Self-Testing on Testing Promotion and Risk Behavior Reduction Among Transgender Women in China: Randomized Controlled Trial

**DOI:** 10.2196/58591

**Published:** 2024-10-29

**Authors:** Yan-Yan Zhu, Ze-Hao Ye, Zhen-Xing Chu, Yingjie Liu, Jie Wei, Le Jia, Yong-Jun Jiang, Hong Shang, Qing-Hai Hu

**Affiliations:** 1 State Key Laboratory for Diagnosis and Treatment of Infectious Diseases NHC Key Laboratory of AIDS Prevention and Treatment, National Clinical Research Center for Laboratory Medicine The First Hospital of China Medical University, China Medical University Shenyang China; 2 Key Laboratory of AIDS Immunology Chinese Academy of Medical Sciences Shenyang China; 3 Key Laboratory of AIDS Immunology of Liaoning Province Shenyang China; 4 Ningbo Municipal Centre for Disease Control and Prevention Ningbo China

**Keywords:** HIV, HIV self-testing, testing behavior, sexual behaviours, transgender women, sexual health, mobile phone

## Abstract

**Background:**

To date, no randomized controlled trials have specifically addressed behavior changes after HIV self-testing (HIVST) among transgender women.

**Objective:**

This study aims to evaluate the effects of HIVST on changes in HIV testing behavior, frequency of condomless sex, and partner numbers among transgender women in China.

**Methods:**

Participants were recruited from 2 Chinese cities using both online and offline methods. Transgender women were randomly assigned to receive an HIVST intervention. Data from the previous 3 months were collected at baseline, 3 months, and 6 months. The primary outcome was the mean change in the number of HIV tests among transgender women during the 6-month follow-up. An intention-to-treat analysis was conducted. The statistical analysis used analysis of covariance and linear mixed-effects models.

**Results:**

From February to June 2021, and 255 transgender women were recruited, of which only 36.5% (93/255) had a steady job, and 27.1% (69/255) earned less than US $414.9 of income per month. They were randomly assigned to the intervention (n=127) and control (n=128) groups. At 6 months, the mean number of HIV tests was 2.14 (95% CI 1.80-2.48) in the intervention group and 1.19 (95% CI 0.99-1.40) in the control group (P<.001), with increases of 0.84 (95% CI 0.54-1.14) and 0.11 (95% CI –0.19-0.41) over 6 months, respectively. The net increase was 0.73 (95% CI 0.31-1.15; P<.001), with a similar adjusted result. No significant differences in the frequency of condomless sex or partner numbers were observed between the 2 groups.

**Conclusions:**

HIVST is an effective strategy for enhancing regular HIV testing behavior among transgender women in China. This strategy should be combined with measures to address the financial vulnerability of the transgender women community to reduce subsequent risk behaviors, including condomless sex.

**Trial Registration:**

Chinese Clinical Trial Registry ChiCTR2000039766; https://www.chictr.org.cn/showproj.html?proj=61402

## Introduction

Transgender women are individuals assigned male at birth who identify as female and have a disproportionate demographic burden of the HIV worldwide; a meta-analysis encompassing data from 15 countries yielded an estimated HIV prevalence of 19.1% among transgender women, with an OR of 48.8 for HIV infection relative to the general population [1]. Data collated between 2018 and 2022 from 9 countries in Asia and the Pacific indicated a median HIV prevalence among transgender women of 3.9%, which was 19.5 times higher than that among general adults (0.2%) [2]. Notably, a study from Shenyang, China, found an HIV prevalence of 24.7% among transgender women [3], significantly exceeding that of other key populations, such as men who have sex with men (MSM) in China [4]. Minority stress and adverse mental health conditions frequently lead transgender women to engage in condomless sex with both regular and casual partners [5-7], elevating their risk of HIV transmission. HIV testing is the gateway to HIV prevention and treatment services; however, transgender women often encounter considerable obstacles to accessing HIV testing services. In addition to financial problems [8], issues of confidentiality, stigma, and discrimination by health care providers in facility-based HIV testing settings are prevalent [9-12], thereby hindering the use of HIV testing and related health care services by transgender women and obstructing efforts to eradicate AIDS.

To achieve the UNAIDS 95-95-95 targets for HIV [2], strategies must prioritize transgender women, who exhibit higher rates of prevalence and transmission risk. HIV self-testing (HIVST) offers greater convenience and privacy than conventional facility-based testing, mitigating stigma and discrimination from health care providers [13]. Randomized controlled trials (RCTs) have confirmed the efficacy and safety of HIVST in scaling up testing services, primarily among MSM and their partners [14-16]. The World Health Organization endorsed HIVST in 2016 to expand testing services [17], which were subsequently incorporated into the *National Guidelines for Detection of HIV/AIDS* in China [18]. Despite the high acceptability of HIVST among transgender women [19,20], high-quality evidence is needed to assess its impact on HIV testing behaviors.

Furthermore, studies have reported inconsistent findings on sexual behaviors and partner numbers post HIVST among MSM and female sex workers [21-23]. A Chinese RCT noted a marked reduction in sexual partners following HIVST among MSM but no change in condomless sex [16]. Research on the post-HIVST behavioral changes among transgender women is scant, with only 1 qualitative study yielding inconclusive results regarding condom use [24]. Additional research is needed to investigate the role of HIVST in health promotion strategies.

We conducted this RCT to evaluate the effects of HIVST on regular HIV testing behaviors among transgender women in China over 6 months. Secondary objectives included assessing the effect of HIVST on the frequency of condomless sex and partner numbers.

## Methods

### Study Design and Participant Eligibility

An open-label RCT was conducted at 2 sites in China (Shenyang and Beijing), using both online and offline recruitment methods (the distribution of 255 transgender women is presented in Figure S1 in Multimedia Appendix 1). The first participant was enrolled in this trial on February 25, 2021, and the last on June 24, 2021. This trial was ended on January 5, 2022. Digital informed consent was obtained from all participants. The trial protocol (Multimedia Appendix 2) was approved by the ethics committee of the First Hospital of China Medical University in Shenyang and adhered to the CONSORT (Consolidated Standards of Reporting Trials) 2010 guidelines as detailed in Multimedia Appendix 3.

We recruited transgender women through community-based organizations (CBOs) at both study sites. An initial online questionnaire screened potential participants referred by CBOs, as well as 10 preconfirmed transgender women. The inclusion criteria were age between 18 and 65 years, assigned male at birth, and self-identification as female. Verification of transgender women identity included responses to specific questions and, when necessary, photographic evidence in women’s attire. Eligible candidates also reported proficiency in using smartphones and engagement in sexual activity within the past 6 months. The exclusion criteria encompassed a positive HIV status before enrollment and refusal to participate in this trial. All qualifying HIV-negative transgender women were subsequently admitted as participants. Throughout this trial, stringent measures were used to preserve the confidentiality of participants’ personal information. All participants retained the right to withdraw from this trial at any point without incurring any negative repercussions.

### Randomization and Masking

We simply randomized participants to either the intervention or control group (1:1). Random numbers were generated using the RANDBETWEEN function in Microsoft Excel 2021 and subsequently concealed with sequentially numbered, opaque envelopes. The investigator opened these envelopes immediately before allocation. Each participant received a unique identification number based on their sequence of enrollment (1-128) and group assignment, with numbers in the intervention group and the control group commencing at “500” and “600”, respectively (eg, 500007 and 600007). Due to the nature of the distinct interventions, blinding of participants was not feasible.

### Procedures

Participants were instructed to add a WeChat account created specifically for this trial. Candidates were asked to complete an online baseline questionnaire distributed by study staff through the dedicated WeChat account and undergo HIVST we provided to exclude those with positive results, who were then referred for confirmatory testing and HIV care. To enhance the acceptability of the questionnaire, we invited 10 transgender women to revise sensitive content and inappropriate expressions before this trial.

After randomization, participants assigned to the intervention group could apply for 1 to 5 complimentary quadruplex self-testing kits from Wondlfo, Guangzhou, China (details are described in Multimedia Appendix 2), both at baseline and at the 3-month follow-up. Each kit had an attached label containing the participant’s unique identification number, HIVST kit number, and a QR code for anonymous result feedback. HIVST kits and instruction cards were packaged without any personal or HIV-related information. We also sent operation instructions through WeChat, including online support for any inquiries. Participants could report their HIVST results by scanning the QR code and uploading photos of the test and label. Participants in both the intervention and control groups attended follow-up visits at 3 and 6 months; during this trial, participants received regular HIV-related health information (eg, free HIV testing locations, window period for HIV seroconversion, benefits of regular HIV testing). Condoms and lubricants were offered to all participants at no cost. Confirmatory testing at clinics was arranged for individuals with positive HIVST kit results; those with confirmatory positive results were linked to HIV care.

Participants were followed up online at 3-month intervals, and online surveys were conducted to assess their HIV testing experiences and related risk behaviors during the previous 3 months. Follow-up questionnaires were disseminated through the dedicated WeChat account. During follow-up, the identity of participants was confirmed using the unique identification number assigned to each individual at the time of enrollment. Participants were asked to upload an image of their test results within 48 hours using the QR code provided for feedback. Reminder messages were sent to participants who failed to upload their results within 1 week. Participants who failed to upload their results within 2 weeks became ineligible to request additional kits. Each participant received financial compensation of US $6.9 for completing each follow-up survey, with an additional US $1.4 awarded for the successful upload of a test result photo.

### Outcomes

We recommended that individuals at risk of HIV have regular HIV testing every 3 months. At the 3- and 6-month follow-ups, we collected the number of HIV tests over the past 3 months to evaluate the behaviors associated with regular HIV testing. The primary outcome was the mean changes in the number of HIV tests among transgender women during 6 months of follow-up, which included both HIVST and facility-based HIV testing. The secondary outcomes included the mean changes in the number of HIV tests during 3 months of follow-up, the mean changes in the frequency of condomless sex, and the number of partners during 6 months of follow-up. The results were self-reported by transgender women. Data were collected through online questionnaires at baseline and at the 3- and 6-month follow-ups, including sociodemographic characteristics, perception of HIVST, number of sexual partners, sexual behaviors, and frequency of HIV testing in the past 3 months. We reminded transgender women by WeChat text messages to complete follow-up questionnaires.

### Statistical Analysis

We used PASS (version 15.0; NCSS, LLC) to calculate the sample size based on a repeated measures design with 2 time points. Enrollment of 103 participants per group was projected to provide 90% power to detect a mean difference of 0.35 in the number of HIV tests per 3 months [15] between groups (2-tailed α=.05). At least 115 patients per group were needed assuming an anticipated 10% dropout rate.

An intention-to-treat analysis was conducted, in which outcomes were compared between participants according to their randomization assignment rather than their actual application for the intervention. To evaluate the effect of HIVST on mean changes in the number of HIV tests among transgender women (primary outcome) and their partner number and frequency of condomless sex (secondary outcome), we compared the outcome variables between the 2 groups. We also evaluated the number of HIV tests by recoding the number of tests into 2 binary variables: yes or no.

Net differences in mean changes from baseline in outcome variables between groups were analyzed using covariance analysis, with the group as a factor and baseline value as a covariate. Adjusted net differences were adjusted for important covariables, including age, ethnicity, education, occupation, income, marital status, transient population, and baseline value (HIV test number, HIV testing history, partner number, or sexual behavior number). A step-down approach was used to find a significant result; the primary and secondary outcomes were tested sequentially to control the familywise type I error rate according to the following order: mean changes in the number of HIV tests at 6 months (primary outcome), mean changes in the number of HIV tests at 3 months, mean changes in the sexual partner number at 6 months, and mean changes in the frequency of condomless sex at 6 months. Statistical significance (2-sided *P*<.05) at each step was required to test the next hypothesis. We used a linear mixed-effects model with individuals as a random effect to evaluate the main effects of the intervention and interaction effects of time on the primary outcome.

Subgroup analyses for the primary outcome were performed based on age, education, occupation, income, marital status, and transient population. For sensitivity analysis, the results with imputation are explored. We assumed that data were missing completely at random and imputed the number of HIV tests, the number of sexual behaviors, and the number of sexual partners through predictive mean matching. Similar distributions of variable values before and after imputation are plotted in Figure S2 in Multimedia Appendix 1. All statistical analyses were performed using R software (version 4.2.2; R Foundation for Statistical Computing).

### Ethical Considerations

This trial received approval from the ethics committee of the First Hospital of China Medical University in Shenyang, China (Approval number: (2020)2020-326-2). Participants were provided with digital informed consent and were given a unique identification number for confidentiality.

## Results

### Study Participants and Baseline Characteristics

**Figure 1:**
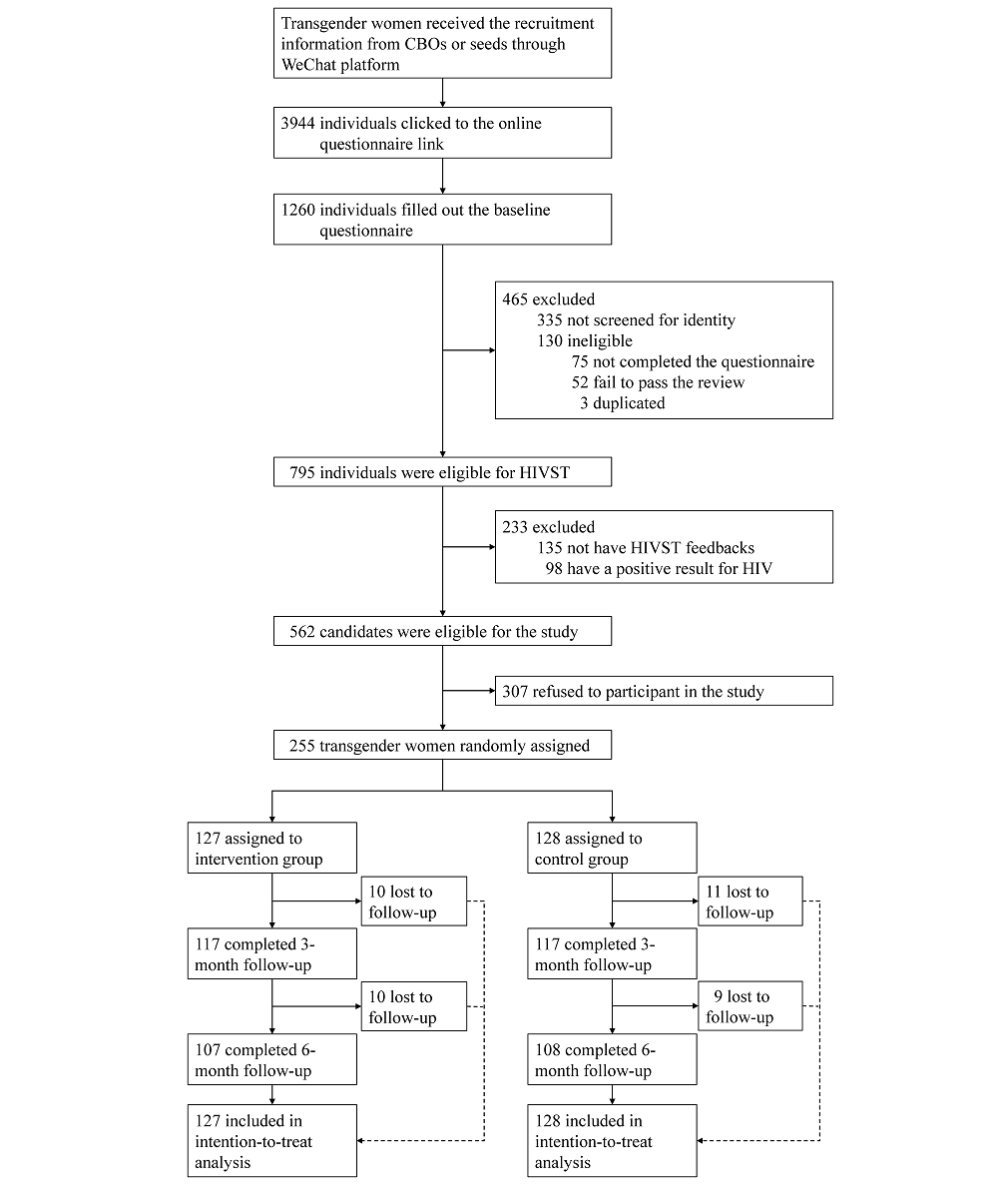
Flow chart of the randomized controlled trial. CBOs: community-based organizations; HIVST: HIV self-testing.

From February 2021 to June 2021, a total of 255 participants who identified as transgender women were enrolled in this trial, with 127 assigned to the intervention group and 128 assigned to the control group (Figure 1). During the 6 months, 20 participants in the intervention group and 20 in the control group were lost to follow-up. The mean age of the participants was 31 (SD 9) years; out of the 255 participants, 140 (54.9%) participants had an education level of college or above, 199 (78%) participants were single, 93 (36.5%) participants had a steady job, and 69 (27.1%) participants earned less than US $414.9 per month. Out of the 255 participants, 180 (70.6%) had a history of HIV testing within the past 3 months, the mean number of HIV tests was 1.2 (SD 1.2), and 30 (11.8%) had a history of pre-exposure prophylaxis uptake (15 in the intervention group). The baseline characteristics of transgender women were similar between the groups (Table 1).

**Table 1:** Demographic and behavioral characteristics of transgender women at baseline.

Characteristics		Intervention (N=127)	Control (N=128)	*P* value	
**Recruitment method, n (%)**					.74
	Facility-based	94 (74)	98 (76.6)		
	Web-based	33 (26)	30 (23.4)		
**Age (years), n (%)**					.88
	18-25	35 (27.6)	37 (28.9)		
	26-35	50 (39.4)	54 (42.2)		
	36-45	30 (23.6)	25 (19.5)		
	≥45	12 (9.4)	12 (9.4)		
**Ethnicity, n (%)**					.73
	Han	112 (88.2)	110 (85.9)		
	Others	15 (11.8)	18 (14.1)		
**Education, n (%)**					.34
	College or above	74 (58.3)	66 (51.6)		
	High school or below	53 (41.7)	62 (48.4)		
**Occupation, n (%)**					.13
	Steady job	53 (41.7)	40 (31.2)		
	Freelancer	31 (24.4)	47 (36.7)		
	Student	20 (15.7)	22 (17.2)		
	Others	23 (18.1)	19 (14.8)		
**Monthly income (US $), n (%)**					.76
	<414.9	36 (28.3)	33 (25.8)		
	414.9-829.6	55 (43.3)	51 (39.8)		
	829.7-1244.5	20 (15.7)	26 (20.3)		
	≥1244.6	16 (12.6)	18 (14.1)		
**Marital status, n (%)**					.45
	Single	95 (74.8)	104 (81.2)		
	Cohabitating	21 (16.5)	15 (11.7)		
	Married	11 (8.7)	9 (7)		
**Transient population, n (%)**					.66
	No	72 (56.7)	77 (60.2)		
	Yes	55 (43.3)	51 (39.8)		
**HIV testing over the past 3 months, n (%)**					.82
	No	36 (28.3)	39 (30.5)		
	Yes	91 (71.7)	89 (69.5)		
HIV test numbers over the past 3 months, Mean (SD)		1.3 (1.3)	1.1 (1.1)	.45	
Partner numbers over the past 3 months^a^, Mean (SD)		6.7 (14.4)	5.9 (12.6)	.61	
Condomless sex numbers over the past 3 months^a^, Mean (SD)		1.8 (4.3)	1.7 (3.3)	.80	

^a^Illogical values (1%) were deleted and imputed with medians.

### Effects Analysis of Primary and Secondary Outcomes

**Figure 2:**
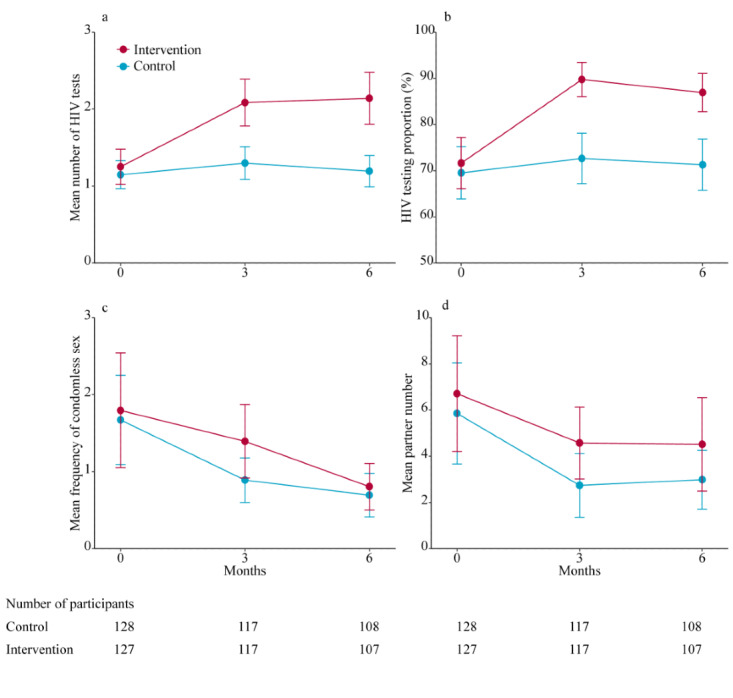
Behavior changes during the 6-month follow-up in the intervention and control groups.

Over 6 months, the number of HIV tests in the intervention group increased by an average of 0.84 tests (95% CI 0.54-1.14) and by 0.11 tests (95% CI –0.19 to 0.41) in the control group (Table 2). The net increase was 0.73 tests (95% CI 0.31-1.15; *P*<.001). After adjustment for potential covariates, this difference remained significant at 0.82 tests (95% CI 0.44-1.19; *P*<.001). At 6 months, the average number of HIV tests was 2.14 (95% CI 1.80-2.48) in the intervention group and 1.19 (95% CI 0.99-1.40) in the control group (*P*<.001; Figure 2A; Table S1 in Multimedia Appendix 1). The main effect of the intervention over the 6 months compared with the control group was an increase of 0.77 tests (95% CI 0.47-1.07; *P*<.001) when the individual was included as a random effect in a linear mixed-effects model. The increase in the number of HIV tests among transgender women was significantly higher in the intervention group than in the control group at 6 months; however, the difference between groups remained stable over time (*P*>.05 for interaction between intervention and follow-up time; Figure 2A).

At 6 months, 93 (86.9%) of 127 transgender women in the intervention group and 77 (71.3%) of 128 transgender women in the control group had been tested for HIV in the previous 3 months, with a group difference of 15.6% (95% CI 4.8-26.4; *P*=.005; Table 2). The proportion of transgender women tested for HIV in the previous 3 months remained steady during the 6-month follow-up (*P*>.05 for interaction between intervention and follow-up time; Figure 2B). None of them were detected with an HIV-positive result. No significant differences were observed in changes in the average number of sexual partners or condomless sex from baseline to 6 months between the 2 groups (Table 2; Figures 2C and 2D).

**Table 2:** Effectiveness of HIV self-testing on outcomes among 255 transgender women.

			Proportion or mean change (95% CI)^a^				Net difference (95% CI)		*P* value		Adjusted net difference^b^ (95% CI)		*P* value
			Intervention		Control								
**Primary outcome**													
	Change in the number of HIV tests at 6 months.	0.84 (0.54 to 1.14)		0.11 (–0.19 to 0.41)		0.73 (0.31 to 1.15)		<.001		0.81 (0.44 to 1.18)		<.001	
**Secondary outcomes**													
	Change in the number of HIV tests at 3 months.	0.82 (0.57 to 1.08)		0.22 (–0.03 to 0.48)		0.60 (0.24 to 0.96)		.001		0.61 (0.28-0.93)		<.001	
	Proportion of HIV testing at 6 months.	86.9% (79.3 to 94.6)		71.3% (63.7 to 78.9)		15.6% (4.8 to 26.4)		.005		13.5% (3.1 to 23.9)		.01	
	Proportion of HIV testing at 3 months.	89.7% (82.8 to 96.7)		72.6% (65.7 to 79.6)		17.1% (7.2 to 27)		<.001		16.8% (7.3 to 26.3)		<.001	
	Change in partner numbers at 6 months.	–2.93 (–5 to –0.87)		–1.85 (–3.91 to 0.21)		–1.08 (–4 to 1.83)		.47		0.45 (–1.52 to 2.41)		.66	
	Change in the frequency of condomless sex at 6 months.	–1.11 (–1.81 to –0.42)		–0.59 (–1.28 to 0.10)		–0.52 (–1.50 to 0.46)		.30		0.05 (–0.34 to 0.44)		.80	

^a^Indicators obtained at every visit through the questionnaire are situations in the previous 3 months (eg, the number of HIV tests in the previous 3 months were obtained at baseline, 3 months, and 6 months, respectively). The change of indicators in the outcomes are all based on values at baseline.

^b^Adjusted for age, ethnicity, education, occupation, income, marital, transient population, HIV testing number, HIV testing history, partner number, or sexual behavior number at baseline.

### Sensitivity Analysis of the Primary Outcome

**Figure 3:**
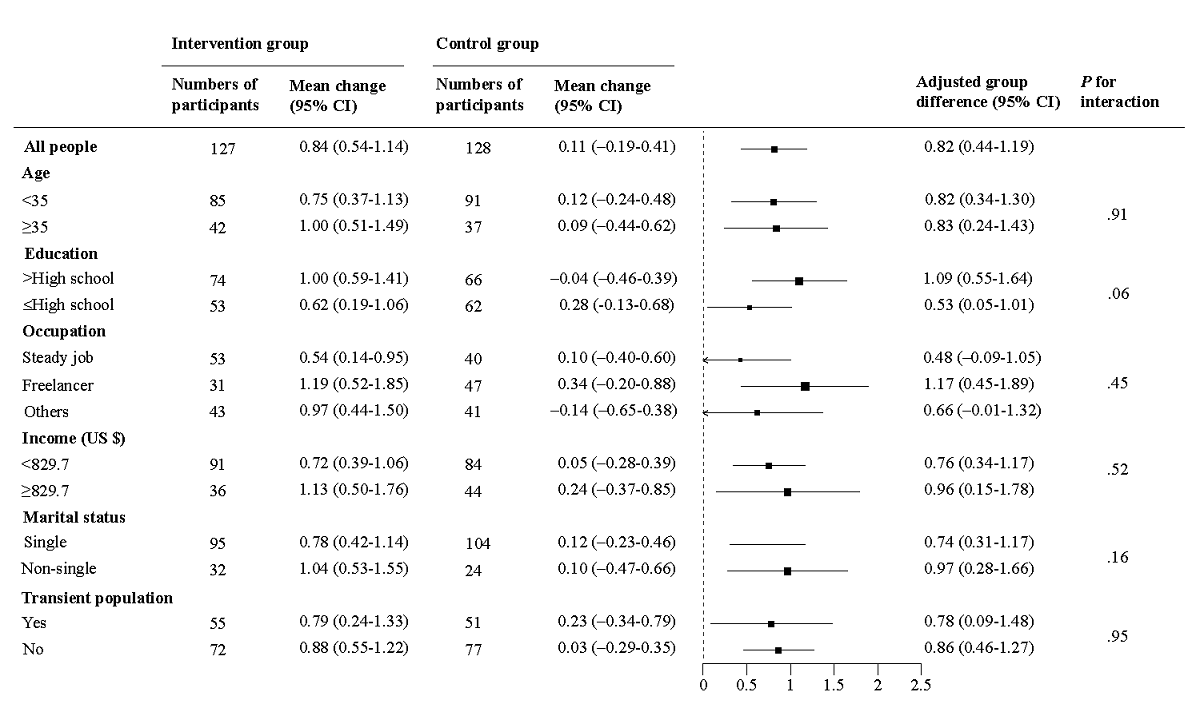
Difference in the mean number of HIV tests at 6 months by subgroup. Error bars indicate 95% CIs.

Mean differences in the number of HIV tests among transgender women between the groups at 6 months remained consistent across subgroups, including age, education, occupation, income, marital status, and transient population (Figure 3). After imputation for missing data, the results for the primary outcome remained consistent; the mean changes in the number of HIV tests among transgender women at 6 months were 0.84 (95% CI 0.56-1.11) in the intervention group and 0.10 (95% CI –0.17 to 0.38) in the control group, with a significant group difference of 0.73 (95% CI 0.34-1.12; *P*<.001; Table S2 in Multimedia Appendix 1).

## Discussion

### Principal Findings

This open-label RCT demonstrated that HIVST was effective in improving HIV testing uptake among transgender women in China. There was a greater mean increase in the number of HIV tests among transgender women in the intervention group compared with those in the control group. A significant net increase in the proportion of HIV testing attributable to HIVST was observed between the 2 groups. However, its reliability as a strategy for reducing risk behaviors is questionable.

### Comparison With Previous Work

In this trial, we observed an almost eightfold difference between the 2 groups in the mean change in the number of HIV tests over 6 months. The intervention led to a notable increase in HIV testing frequency over the previous 3 months (2.14 times per participant), which was greater than that in a trial among MSM in Seattle, Washington [15] and another trial among MSM in Hunan, China [14]; MSM randomized to HIVST reported an average 5.30 times of HIV testing frequency over 15 months (average 1.06 times per participant over 3 months) and an average 3.75 times of HIV testing frequency over 12 months (average 0.94 times per participant over 3 months), respectively. Transgender individuals experience additional disparities compared with their cisgender counterparts, such as increased risk behaviors, stigma, mental health issues, and negative health care experiences (potentially avoiding health care due to fear of mistreatment) [11,25]. This may account for the higher demand among transgender women for HIVST, a method that offers convenience and confidentiality. Similar to previous findings in other populations [16,26], we showed a significantly higher proportion of transgender women in the intervention group who underwent HIV testing. Considering the varied contexts and populations, these studies reported inconsistent effect sizes. Furthermore, the heightened frequency and proportion of regular HIV testing in this trial remained stable at the 3- and 6-month follow-ups, reflecting a sustained high demand for HIV testing among transgender women. Transgender individuals are often left behind in HIV-related programs in Asia and the Pacific [27]. This trial provides further evidence from China that HIVST is effective at establishing and improving regular HIV testing behavior among transgender women.

Despite the effectiveness of HIVST, the cost is one of the main concerns regarding its wider adoption by transgender women [19], particularly among transgender female sex workers [8]. In this trial, 35.7% of transgender women had a steady job and 27.1% had a low-level income. A survey in the United States reported that 19% of transgender individuals have engaged in sex work in their lifetimes and that nearly 29% live in poverty compared with 12% of the general US population [25]. Regular use of HIVST, not to mention partner screening, may be unaffordable for them [24]. Strategies to reduce or subsidize costs are crucial for future HIVST use among transgender women. Linkage to confirmatory testing and subsequent antiretroviral therapy (ART) initiation following a positive HIVST result is another critical challenge. An observational study in Bangkok revealed that nearly half of MSM and transgender women who first tested positive by HIVST failed to start ART [28]. Notably, community-based ART initiation, such as mobile- and home-based platforms, could have the potential to achieve higher ART initiation rates [29,30] and could also facilitate the provision of prevention services (eg, pre-exposure prophylaxis) following HIVST.

Previous studies reported inconsistent results on partner number or frequency of condomless sex following HIVST use [21-23]. In a qualitative study, transgender women reported varied decisions on condom use after receiving negative HIVST results from partners [24]. In this trial, we failed to find a statistically significant reduction in partner number or frequency of condomless sex among transgender women over a 6-month follow-up period. It may be unreliable to consider HIVST as a standalone strategy for promoting condom use among transgender women, as it appears to be more influenced by risk perception [31] and partner type [32].

Transgender women frequently occupy a vulnerable position within partnerships; the reasons for condomless sex include trust in their regular partners and opposition from casual and commercial partners [32]. Discrimination often leads to unemployment among transgender women worldwide, which sometimes makes them involved in high-risk sexual behaviors for income, including having multiple sex partners, condomless sex, and commercial sex [25,27]. Therefore, in addition to increasing the availability of condoms, interventions should address the financial vulnerability of the transgender women community to improve their ability to negotiate condom use within partnerships. For instance, the Avahan program has accomplished this through financial literacy training, assistance with opening savings accounts, and guidance on financial investments [33].

Failure to establish regular HIV testing behavior in key populations will easily lead to missed opportunities for ART initiation and then prolong the pandemic indefinitely, causing the affected community and society to incur substantial costs.

### Strengths and Limitations

In this trial, we first showed the effects of HIVST on improving regular HIV testing behavior and subsequent changes in condomless sex and partner numbers among transgender women in China. However, this trial has several limitations. First, there was potential reporting bias due to self-defined identities among transgender women and the absence of standardized tools for assessing gender identity, as well as our partial reliance on self-reported HIVST results from participants. Second, the mixed online and offline recruitment in this trial may have introduced selection bias from diverse district sources. Furthermore, in this unblinded trial, participant recruitment through CBOs and 10 transgender women seeds may have led to contamination between the intervention and control groups, potentially reducing the difference in intervention effects. Third, most participants recruited offline by 2 CBOs in Shenyang and Beijing may have represented a subpopulation of MSM and engaged in other HIV testing programs, which may have overestimated regular HIV testing behavior among transgender women at baseline. Fourth, the reasons that candidates refused to participate in our trial included no fixed abode, which makes it hard to receive HIVST kits, unwilling to disclose their address, unwilling to participate in regular visits, which may affect their daily lives, and so on; regretfully, we did not systematically collect these causes. Furthermore, understanding why participants did not upload their results would be helpful to inform future approaches to developing research queries for this population. Regretfully, we did not collect specific reasons. Fifth, this trial was conducted during the COVID-19 pandemic. Public health restrictions in China affect the activities of most individuals, including transgender women. Therefore, sexual activity and healthcare access were, to a certain degree, limited among transgender women from both groups. However, the degree of the limitation, we think, is balanced between the 2 groups in this RCT, which will not affect the effect of the intervention we observed between the 2 groups. Finally, our sample presented a regional concentration in Shenyang and Beijing (Figure S1 in Multimedia Appendix 1), limiting the generalizability of our findings.

### Conclusions

This trial demonstrated that HIVST resulted in statistically significant improvements in HIV testing uptake among transgender women in China. This effective intervention strategy should be integrated with actions addressing the financial vulnerability of the transgender women community and strategies for treatment and prevention services to improve condom use and linkages to ART or pre-exposure prophylaxis.

## Appendix

Multimedia Appendix 1 Supplementary tables and figures.

Multimedia Appendix 2 Study protocol.

Multimedia Appendix 3 CONSORT (Consolidated Standards of Reporting Trials) checklist.

## Abbreviations

ART: antiretroviral therapy
CBO: community-based organization
CONSORT: Consolidated Standards of Reporting Trials
HIVST: HIV self-testing
MSM: men who have sex with men
RCT: randomized controlled trial
