# Supplementary material for: Effects of HIV Self-Testing on Testing Promotion and Risk Behavior Reduction Among Transgender Women in China: Randomized Controlled Trial
Source: J Med Internet Res. 2024 Oct 29;26:e58591. doi: 10.2196/58591 (PMC11558219; doi:10.2196/58591)
Supplement: Multimedia Appendix 1 [file jmir_v26i1e58591_app1.docx]

**Table S1. Outcomes of 255 TGW at different time points.**

|  | **Mean or percentage (95% CI)** | |  |
| --- | --- | --- | --- |
|  | **Intervention** | **Control** | ***P* value** |
| **HIV test number** |  |  |  |
| baseline | 1.25 (1.02 to 1.48) | 1.15 (0.97 to 1.33) |  |
| 3-month follow-up | 2.09 (1.78 to 2.39) | 1.30 (1.09 to 1.51) | <0.0001 |
| 6-month follow-up | 2.14 (1.80 to 2.48) | 1.19 (0.99 to 1.40) | <0.0001 |
| **Proportion of HIV testing** |  |  |  |
| baseline | 71.7 (66.1 to 77.2) | 69.5 (63.9 to 75.2) |  |
| 3-month follow-up | 89.7 (86.0 to 93.5) | 72.6 (67.2 to 78.1) | <0.0001 |
| 6-month follow-up | 86.9 (82.8 to 91.1) | 71.3 (65.7 to 76.8) | 0.005 |
| **Partner numbers** |  |  |  |
| baseline | 6.71 (4.21 to 9.21) | 5.85 (3.66 to 8.04) |  |
| 3-month follow-up | 4.57 (3.01 to 6.13) | 2.74 (1.36 to 4.11) | 0.085 |
| 6-month follow-up | 4.51 (2.49 to 6.54) | 2.98 (1.71 to 4.25) | 0.209 |
| **Frequency of condomless sex** |  |  |  |
| baseline | 1.80 (1.05 to 2.54) | 1.67 (1.09 to 2.25) |  |
| 3-month follow-up | 1.39 (0.92 to 1.87) | 0.89 (0.60 to 1.18) | 0.078 |
| 6-month follow-up | 0.80 (0.50 to 1.11) | 0.69 (0.41 to 0.98) | 0.604 |

No imputation was performed for missing data. *P* value was computed by Student’s t test.

See Figure 2 in the article.

**Table S2. Effectiveness of HIVST on outcomes among 255 TGW with imputation by PMM.**

|  | **Proportion or mean change (95% *CI*)** | | **Net difference (95% *CI*)** | ***P* value** | **Adjusted net difference* (95% *CI*)** | ***P* value** |
| --- | --- | --- | --- | --- | --- | --- |
|  | Intervention | Control |  |  |  |  |
| **Primary outcome** |  |  |  |  |  |  |
| Change in the number of HIV tests at 6 months | 0.84 (0.56 to 1.11) | 0.10 (-0.17 to 0.38) | 0.73 (0.34 to 1.12) | 0.0003 | 0.78 (0.44 to 1.11) | <0.0001 |
| **Secondary outcomes** |  |  |  |  |  |  |
| Change in the number of HIV tests at 3 months | 0.76 (0.52 to 1.01) | 0.16 (-0.08 to 0.41) | 0.60 (0.25 to 0.95) | 0.0007 | 0.61 (0.30 to 0.92) | 0.0001 |
| Proportion of HIV testing at 6 months | 86.9% (79.3 to 94.6) | 71.3% (63.7 to 78.9) | 15.6% (4.8 to 26.4) | 0.0047 | 13.5% (3.0 to 24.0) | 0.0116 |
| Proportion of HIV testing at 3 months | 89.7% (82.8 to 96.7) | 72.6% (65.7 to 79.6) | 17.1% (7.2 to 27.0) | 0.0008 | 16.8% (7.3 to 26.3) | 0.0006 |
| Change in partner numbers at 6 months | -2.71 (-4.54 to -0.88) | -2.36 (-4.19 to -0.53) | -0.35 (-2.94 to 2.24) | 0.791 | 0.11 (-1.59 to 1.80) | 0.901 |
| Change in the frequency of condomless sex at 6 months | -1.04 (-1.67 to -0.41) | -0.86 (-1.49 to -0.23) | -0.18 (-1.07 to 0.71) | 0.691 | -0.05 (-0.40 to 0.29) | 0.761 |

Indicators of primary and secondary outcomes are results that occurred in the past 3 months at every visit. *Adjusted for age, ethnicity, education, occupation, income, marital, transient population, and HIV testing number/ partner number/ sexual behavior number at baseline. CI=confidence interval; PMM=predictive mean matching; TGW=transgender women; HIVST=HIV self-testing.


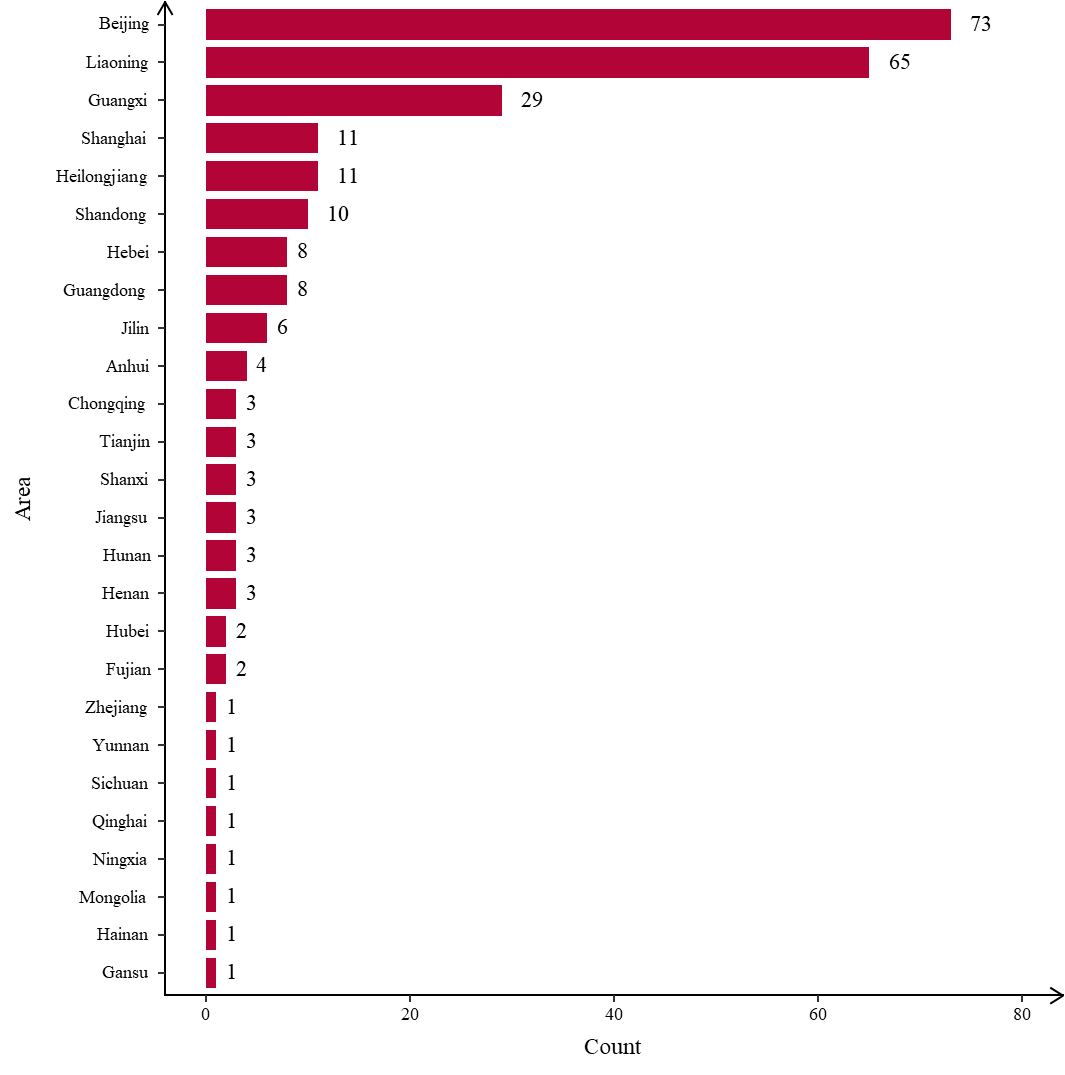


**Figure S1. Address distribution of 255 transgender women.**


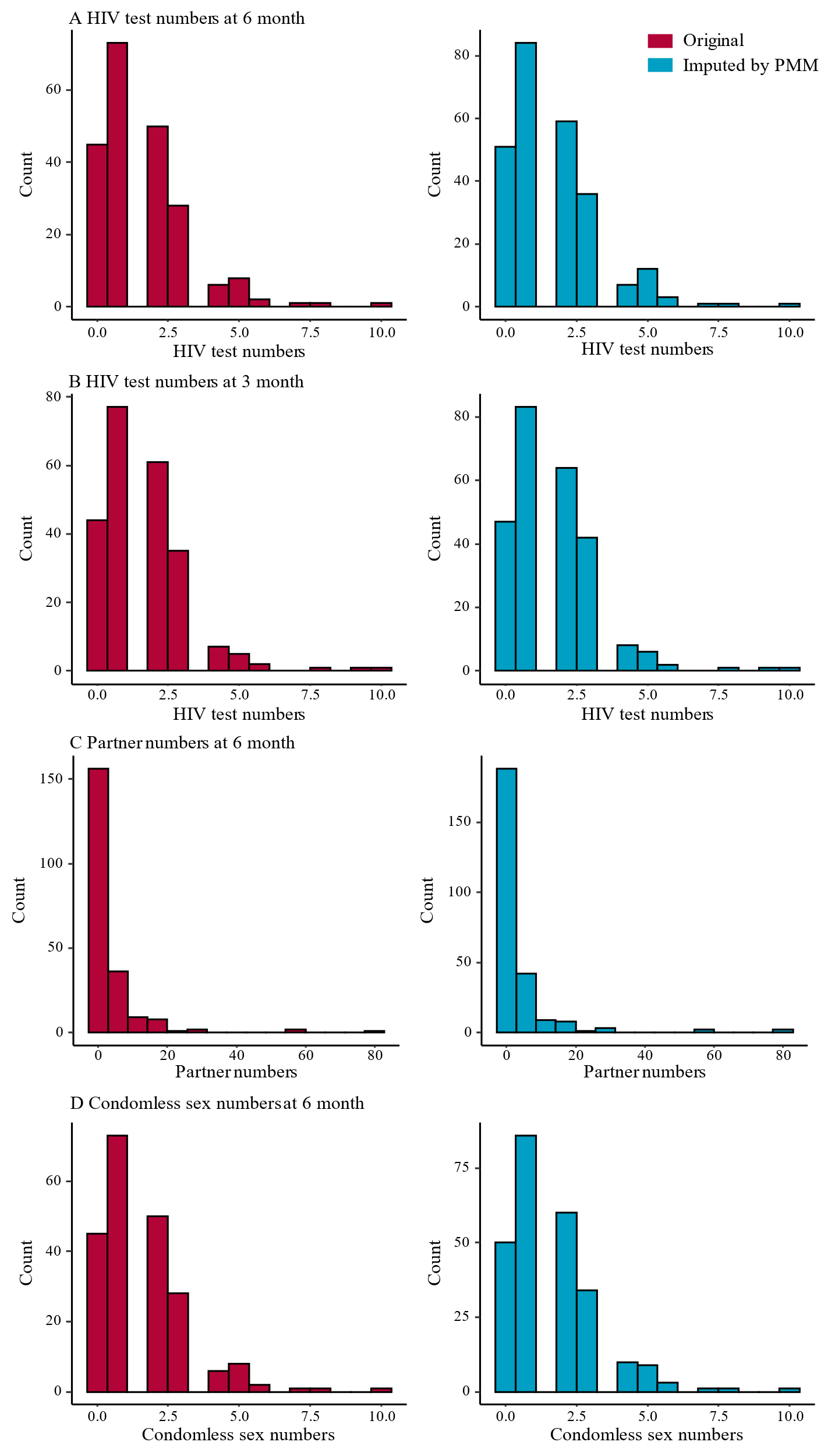


**Figure S2. Comparison on distribution of variable values before and after imputation by PMM.** PMM=predictive mean matching.
