# Supplementary material for: Effects of HIV Self-Testing on Testing Promotion and Risk Behavior Reduction Among Transgender Women in China: Randomized Controlled Trial
Source: J Med Internet Res. 2024 Oct 29;26:e58591. doi: 10.2196/58591 (PMC11558219; doi:10.2196/58591)
Supplement: Multimedia Appendix 2 [file jmir_v26i1e58591_app2.docx]

**Study Protocol**

**Title:** **Effects of HIV self-testing on testing promotion and risk behaviour reduction among transgender women:**

**A randomized controlled trial in China**

**Version 2, Data: September 15, 2020**

Protocol number: 20200915V2.0

Research date: February 2021 - December 2021

Unit of project leader: The First Hospital of China Medical University

Participating unit: Tian Yuan Studio of Beijing You’an Hospital

**Investigator statement**

The clinical trial protocol was developed by the unit of the project leader in collaboration with the investigators. I agree to perform my responsibility as an investigator in accordance with the Declaration of Helsinki and the Quality Management Practice for Pharmaceutical Clinical Trials and to conduct this clinical trial in accordance with the requirements of the clinical trial protocol.

**Unit of project leader: The First Hospital of China Medical University**

Address: No.155 Nanjing North Street, Heping District, Shenyang City, Liaoning Province

Major investigator Name (signature):____________ Date:__________

Telephone: ____________

Fax: ____________

**Participating unit: Tian Yuan Studio of Beijing You’an Hospital**

Address: No. 8, Xitoutiao, Right Anwai, Fengtai District, Beijing

Major investigator Name (signature):____________ Date:__________

Telephone: ____________

Fax: ____________

TABLE OF CONTENTS

[**Protocol Synopsis** 7](#_Toc151649712)

[**Study Glossary** 11](#_Toc151649713)

[**1. OBJECTIVES** 12](#_Toc151649714)

[**1.1 Primary Objective** 12](#_Toc151649715)

[**1.2 Secondary Objectives** 12](#_Toc151649716)

[**2. BACKGROUND AND RATIONALE** 12](#_Toc151649717)

[**2.1 Hypotheses** 14](#_Toc151649718)

[**3. IMPLEMENTATION PLAN** 14](#_Toc151649719)

[**3.1 Study Design** 14](#_Toc151649720)

[**3.2 Number of Study Sites** 15](#_Toc151649721)

[**3.3 Replacement of Participants** 15](#_Toc151649722)

[**3.4 Estimated Study Duration** 15](#_Toc151649723)

[**3.4.1 Study Duration for Participants** 15](#_Toc151649724)

[**4. PARTICIPANTS' ELIGIBILITY** 16](#_Toc151649725)

[**4.1 Inclusion and Exclusion Criteria** 16](#_Toc151649726)

[**4.1.1 Inclusion Criteria** 16](#_Toc151649727)

[**4.1.2 Exclusion Criteria** 17](#_Toc151649728)

[**5. RANDOMIZATION AND MASKING** 17](#_Toc151649729)

[**6. PARTICIPANTS ENROLLMENT** 17](#_Toc151649730)

[**7. STUDY PROCEDURES** 18](#_Toc151649731)

[**7.1 Baseline** 18](#_Toc151649732)

[**7.2 Follow Up** 18](#_Toc151649733)

[**7.3 Intervention Contents** 18](#_Toc151649734)

[**7.3.1 Quadruplex Self-Testing Package** 18](#_Toc151649735)

[**7.4 Intervention Procedures** 19](#_Toc151649736)

[**7.5 Measures of Different HIV Testing Results** 20](#_Toc151649737)

[**7.6 Questionnaire Contents** 20](#_Toc151649738)

[**8. WITHDRAWAL FROM THE STUDY** 21](#_Toc151649739)

[**8.1 Decision to Withdraw from Participants or Investigators** 21](#_Toc151649740)

[**8.2 Reasons for Removal from Study** 21](#_Toc151649741)

[**9. ADVERSE EVENTS** 21](#_Toc151649742)

[**10. STATISTICAL CONSIDERATIONS** 21](#_Toc151649743)

[**10.1 Intention-to-Treat Set (ITT) Population** 21](#_Toc151649744)

[**10.2 General Considerations** 22](#_Toc151649745)

[**10.3 Efficacy Analysis** 22](#_Toc151649746)

[**10.3.1 Outcome Measure** 22](#_Toc151649747)

[**10.3.2 Analysis of the Primary and Secondary Efficacies** 22](#_Toc151649748)

[**10.3.3 Sensitivity analysis of the primary intervention effect** 23](#_Toc151649749)

[**10.4 Sample Size Considerations** 23](#_Toc151649750)

[**10.4.1 Tests for Two Means in a Repeated Measures Design in PASS** 23](#_Toc151649751)

[**10.4.2 Summary Statements** 24](#_Toc151649752)

[**11. QUALITY CONTROL** 25](#_Toc151649753)

[**11.1 Design Phase of the Protocol** 25](#_Toc151649754)

[**11.2 Implementation Phase of the Protocol** 25](#_Toc151649755)

[**11.3 Analysis Phrase of Data** 25](#_Toc151649756)

[**12. ETHICS** 26](#_Toc151649757)

[**12.1 Informed Consent** 26](#_Toc151649758)

[**12.2 Institutional Review Board/Independent Ethics Committee** 26](#_Toc151649759)

[**12.3 Confidentiality** 26](#_Toc151649760)

[**13. DATA PROCESSING AND STORAGE** 27](#_Toc151649761)

[**13.1 Database Establishment** 27](#_Toc151649762)

[**13.2 Data Storage** 27](#_Toc151649763)

[**13.3 Data Availability** 27](#_Toc151649764)

[**14. TIMELINE OF STUDY PLAN** 27](#_Toc151649765)

[**15. ROLE OF THE FUNDING SOURCE** 28](#_Toc151649766)

[**16. DECLARATION OF INTERESTS** 28](#_Toc151649767)

[**17. REFERENCE** 28](#_Toc151649768)

[**Appendix A. Informed Consent** 30](#_Toc151649769)

[**Appendix B. Screening questionnaire**  35](#_Toc151649770)

[**Appendix C. Online questionnaire (partly)** 36](#_Toc151649771)

**Protocol Synopsis**

Title: Effects of HIV self-testing on testing promotion and risk behaviours reduction among transgender women: a randomized controlled trial in China

**Primary Objective**

To evaluate the efficacy of the intervention (HIV self-testing, HIVST) in increasing HIV test numbers among transgender women (TGW) during six months of follow-up.

**Secondary Objectives**

- To evaluate the efficacy of HIVST in increasing HIV tests numbers during three months of follow-up.
- To evaluate the efficacy of HIVST in increasing HIV testing proportion during six months of follow-up.
- To evaluate the efficacy of HIVST in increasing HIV testing proportion during three months of follow-up.
- To evaluate the efficacy of HIVST in changing the partner numbers during six months of follow-up.
- To evaluate the efficacy of HIVST in changing the frequency of condomless sex during six months of follow-up.

**Hypothesis**

The primary hypothesis is that HIVST can improve regular HIV testing behavior among TGW.

**Primary Outcome:** The mean changes in the number of HIV tests among TGW during six months of follow-up.

**Secondary Efficacy Outcomes:**

- The mean changes in the number of HIV tests during three months of follow-up.
- The proportion of HIV testing at six months.
- The proportion of HIV testing at three months.
- The mean changes in partner numbers during six months of follow-up.
- The mean changes in the frequency of condomless sex during six months of follow-up.

**Study Design:** This study is an open-label, randomized controlled trial. Eligible participants for screening are HIV-negative TGW. The minimum expected total recruitment of TGW is approximately 230, with a minimum expected recruitment of approximately 115 participants in the intervention group and the control group.

**Sample Size:** We used PASS V. 15.0 (NCSS, LLC. Kaysville, Utah, USA) to calculate the sample size based on a repeated measures design with two time points. Enrolment of 103 participants per group was projected to provided 90% power to detect a mean difference of 0.35 in the number of HIV tests per three months^1^ between groups (two-tailed α = 0.05). At least 115 patients per group were needed assuming an anticipated 10% dropout rate.

**Summary of Participants Eligibility Criteria:** The following are inclusion criteria for candidates: being 18-65 years of age, assigned male at birth and self-identifying as female, no previous record of HIV positive test results, tested negative for HIV antibodies in the rapid test during the screening, engaged in sexual activity in the past six months, willing to provide truthful information about their sexual behaviour, own a smartphone and able to use WeChat, and provide electronic informed consent. The following are the exclusion criteria for candidates: candidates who do not meet the defined inclusion criteria, encompassed a positive HIV status prior to enrolment and refusal to partake in the study.

**Intervention Contents:**

**Quadruplex self-testing package:** The quadruplex self-testing kits to be used (Wondfo Guangzhou, China) are finger stick-based, which can detect HIV-1 antibody, Treponema pallidum antibody, Hepatitis B surface Antigen, and Hepatitis C virus antibody in blood specimens at 6 weeks after infection. The kits will be packaged with the required consumables during finger blood sample collection (a disposable retractable safety lancets, alcohol pad, capillary tube, Wondlfo solution, and a sealed bandage). Each package will contain corresponding identification numbers and instructions for the testing procedure, results interpretation, and results upload.

**Procedures:**

After including TGW who test negative for HIV and meet the eligibility criteria, they will be randomly assigned to the intervention group and the control group. Based on participants' needs, the intervention group will have the opportunity to apply for free HIV self-testing (HIVST) kits and mailing services every three months (via WeChat). Participants can request to receive 1-5 self-testing kits based on their own needs. HIV-related health information will be regularly sent to both the intervention and control groups at predetermined intervals (at enrolment and three months after enrolment). Follow-up surveys will be conducted, and electronic questionnaires will be filled out at three months and six months after enrolment. To protect personal privacy, all HIV self-testing kits mailed to participants will be discreetly packaged in an unmarked box along with the instructions. Each self-testing kit will be assigned a unique code for future tracking and result retrieval. Users of the self-testing kits can scan the QR code on the instructions, take a photo of the self-test result, and anonymously upload it to the online platform (created through the online questionnaire platform: https://jinshuju.net/home).

**Statistical Considerations:**

**General Considerations**

A summary of the demographic and behavioural characteristics of the participants will be provided. Descriptive statistics for continuous variables will include the mean and standard deviation (if they follow a normal distribution) or the median and interquartile range (if they do not follow a normal distribution). For categorical variables, frequencies and percentages will be reported.

The Intention-to-Treat Set (ITT) is defined as all randomized participants. All efficacy analyses will be performed based on the ITT population.

**Analysis of the Primary and Secondary Efficacies**

Net differences in mean changes from baseline in outcome variables between groups will be analysed using covariance analysis, with group as a factor and baseline value as a covariate. A step-down approach will be used to find a significant result; the primary and secondary outcomes are to be tested sequentially to control the familywise type I error rate according to the following order: mean changes in the number of HIV tests at six months (primary outcome), mean changes in the number of HIV tests at three months, mean changes in the sexual partner number at six months, and mean changes in the frequency of condomless sex at six months. Statistical significance (two-sided P<0.05) at each step is required to test the next hypothesis. Linear mixed-effects model with individuals as a random effect will be used to evaluate the main effects of the intervention and interaction effects of time on the primary outcome. Statistical analysis will be conducted using R software. For a detailed description of the statistical analysis methods, please refer to Section 10.

**Study Glossary**

| Abbreviation or Term | Definition/Explanation |
| --- | --- |
| CBOs | Community-Based Organizations |
| CI | Confidence Interval |
| HIV | Human Immunodeficiency Virus |
| HIVST | Human Immunodeficiency Virus Self-Testing |
| ITT | Intention-To-Treat Analysis Set |
| QR | Quick response |
| RCT | Randomized Controlled Trial |
| TGW | Transgender Women |

**1. OBJECTIVES**

**1.1 Primary Objective**

To evaluate the efficacy of the intervention (HIV self-testing, HIVST) in increasing HIV test numbers among transgender women (TGW) during six months of follow-up.

**1.2 Secondary Objectives**

- To evaluate the efficacy of HIVST distribution in increasing the mean changes in the number of HIV tests during three months of follow-up.
- To evaluate the efficacy of HIVST in increasing HIV testing proportion during six months of follow-up.
- To evaluate the efficacy of HIVST in increasing HIV testing proportion during three months of follow-up.
- To evaluate the efficacy of HIVST in changing the partner numbers during six months of follow-up.
- To evaluate the efficacy of HIVST in changing the frequency of condomless sex during six months of follow-up.

**2. BACKGROUND AND RATIONALE**

The term "transgender" refers to individuals whose gender identity and expression differ from the sex they were assigned at birth, regardless of whether they have undergone medical interventions such as gender-affirming surgeries, hormone therapy, facial reconstruction, etc. Within the transgender population, there are two main categories based on self-identified gender: transgender women (TGW) and transgender men (TGM). TGW refers to individuals who were assigned male at birth but identify and express themselves as female^2^. TGW are a key population for HIV infection globally. A meta-analysis of HIV infection among transgender women worldwide showed an average HIV prevalence rate of 19.1%. The risk of HIV infection among transgender individuals is 48.8 times higher compared to the general population^3^. However, the extreme sense of shame, discrimination, and fear of positive results often experienced by transgender individuals often leads to their reluctance to seek healthcare or social services^4^, resulting in lower HIV testing rates^5,6^. To achieve the goal of ending the HIV/AIDS epidemic by 2030, there is an urgent need to optimize the HIV prevention and treatment strategies for TGW at present.

Globally, only 65.3% of the TGW has undergone HIV testing, with 66.6% of TGW in the United States being tested for HIV ^6^. In China, only 34.6% of the TGW has undergone HIV testing ^7^. In some low- and middle-income countries, the proportion of HIV-tested TGW is less than 50%, such as 27.0% in Brazil, 43.6% in Thailand, and 43.9% in Vietnam^8-10^. The proportion of TGW aware of their HIV infection status is still far from the first 90% target, highlighting the urgent need to explore effective strategies to expand testing and meet the testing needs of the transgender population^2^ .

HIV self-testing (HIVST) offers convenience, privacy, and ease of use^11^. It eliminates some typical barriers to testing, such as inconvenience (related to transportation, time, and location), privacy concerns, and stigma^12^, providing TGW with a non-discriminatory opportunity for HIV testing. The use of HIVST is supported by the World Health Organization, particularly for populations with low healthcare service coverage, high risk of HIV infection, and limited access to HIV testing services^13^. Previous studies have found that HIVST not only increases testing frequency among men who have sex with men (MSM)^14^, but also increases testing frequency among male truck drivers^15^, female sex workers^16^, and women and their male partners seeking antenatal care^17^. HIVST has shown high acceptance rates among TGW and has facilitated HIV testing among their sexual partners and those who have never been tested before^18-21^.

While global health organizations, including the World Health Organization, recognize transgender individuals as a distinct and special population, many researchers consider TGW as a subgroup of men who have sex with men (MSM). Epidemiological surveys often combine MSM and TGW regardless of gender identity and sexual orientation, which may blur how TGW acquire HIV and whom they may transmit the infection to^22^. Studies have shown that the sexual networks of TGW and their partners may differ from those of MSM, and TGW have different characteristics compared to cisgender men who have sex with men^23^. Therefore, there is currently a lack of urgently needed interventions specifically targeting TGW, such as HIV testing promotion programs^24^.

In summary, HIVST has shown good acceptance and significant potential for development in the TGW due to its convenience, confidentiality, and privacy. However, there is a lack of randomized controlled studies both domestically and internationally that utilize HIVST to promote HIV testing among TGW.

**2.1 Hypotheses**

We hypothesized that HIVST can improve regular HIV testing behavior among TGW.

**3. IMPLEMENTATION PLAN**

**3.1 Study Design**

This study is an open-label, randomized controlled trial. Participants who meet the inclusion criteria are recruited in Shenyang and Beijing through a combination of online and offline methods. Participants are randomly assigned in a 1:1 ratio to either the intervention group or the control group. The researchers provide the intervention group TGW with a link to apply for HIV self-testing kits, with each participant being able to request 1-5 kits as needed. The application link is provided only at the time of enrolment and at three months after enrolment. The researchers conduct electronic questionnaire surveys with the participants at baseline, three months after enrolment, and six months after enrolment to analyse the impact of HIV self-testing on HIV testing behaviour among TGW.

The overall study design is described by a flow chart as follows (Figure 1):


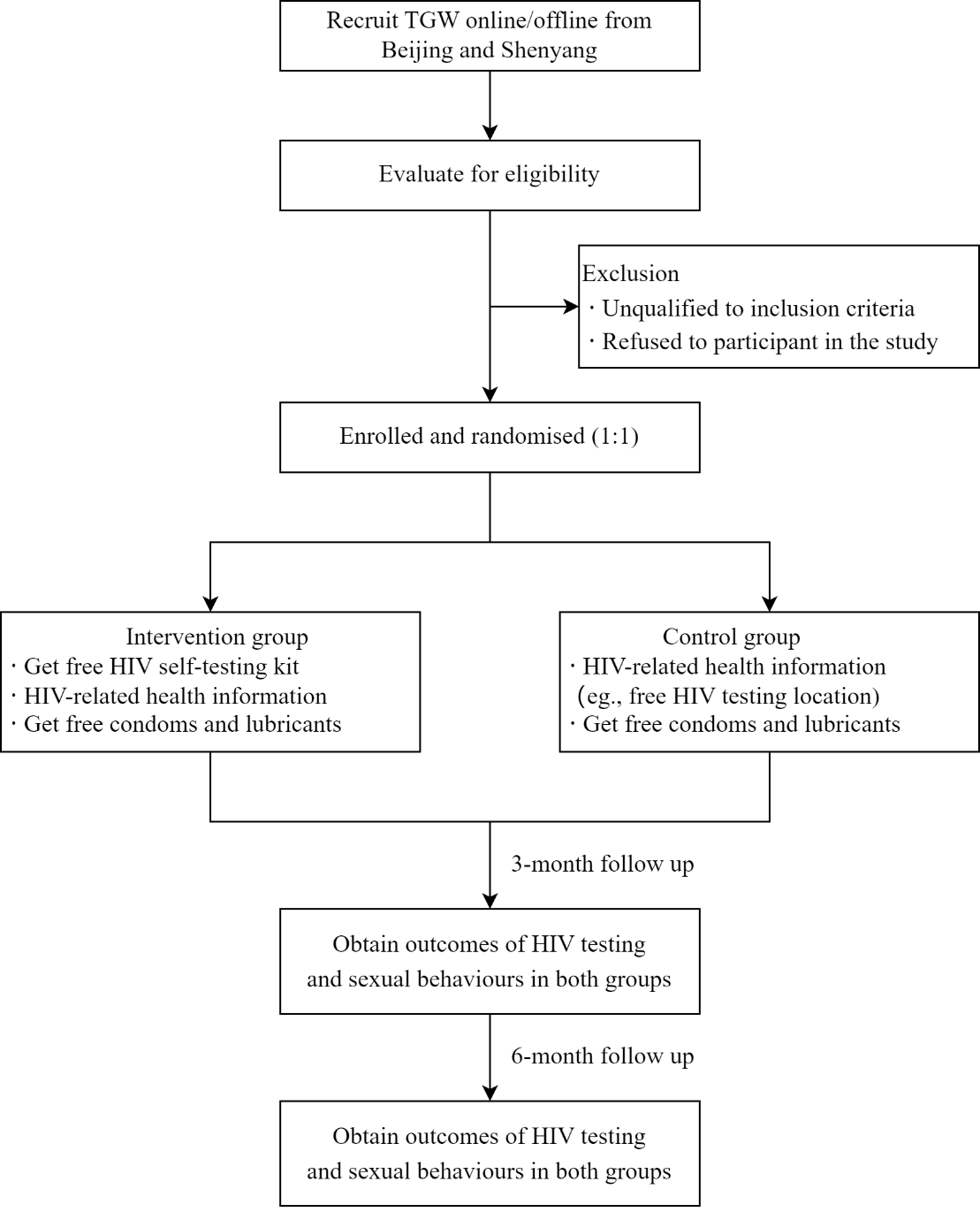


**Figure 1 Flow chart of study design**

**3.2 Number of Study Sites**

This study is an online intervention survey research that will recruit participants in two cities in northern China, Shenyang and Beijing.

**3.3 Replacement of Participants**

There will be no replacement for participants.

**3.4 Estimated Study Duration**

**3.4.1 Study Duration for Participants**

The plan is to recruit participants from January 2021 to June 2021. For all participants involved in the study, the duration of the study after enrolment is six months.

**3.4.2 End of Study**

Primary Completion: The primary completion date refers to the point at which the last participant is assessed or receives intervention for the final collection of data for the primary endpoint(s), whether the study concluded as planned in the protocol or was terminated early.

The primary completion date is the same as the end-of-study date and is the date when the last enrolled participant has completed the end-of-study visit (month 6) (i.e., the last participant's last visit).

If the study concludes prior to the primary completion date originally planned in the protocol (i.e., early termination of the study), then the primary completion date corresponds to the last subject’s last visit.

End of Study: The end of the study date is defined as the last enrolled participant’s completion of the end-of-study visit (month 6) or terminates of the study early. Terminating early means that enrolled participants complete the study visit ahead of schedule.

**4. PARTICIPANTS' ELIGIBILITY**

Investigators will be required to have a screening log of all potential study candidates that include limited information about the potential candidate, such as the screening date. This log can be completed and updated through an online survey platform (https://jinshuju.net/home).

Before conducting any research-related procedures, appropriate digital informed consent (and, where applicable, assent) must be obtained (see Section 12.1).

**4.1 Inclusion and Exclusion Criteria**

**4.1.1 Inclusion Criteria**

- Aged 18 to 65 years old.
- Assigned male at birth, and self-identifying as female.
- Tested negative for HIV antibodies during the screening at enrolment.
- Engaged in sexual activity in the past six months.
- Willing to provide truthful feedback on their sexual behaviour.
- Owns a smartphone and is able to use WeChat.
- Willing to sign an informed consent form.

**4.1.2 Exclusion Criteria**

- Unwilling to participate in follow-up.
- Encompassed a positive HIV status prior to enrolment.
- Refused to use HIV self-testing (HIVST).

**5. RANDOMIZATION AND MASKING**

Randomization will be performed based on a computer-generated random sequence using SPSS software version 26.0 (SPSS, IBM Inc) with a random seed of 230. The sequence will be divided into two groups by the Visual Binning function. Enrolment will be ongoing during the trial period. Due to the different intervention approaches between the two groups, blinding was not feasible for CBO staff and participants.

**6. PARTICIPANTS ENROLLMENT**

Before initiating specific research activities/procedures, all participants must personally sign the digital informed consent form. Each participant has been assigned a unique identification number, which will be used throughout the study to identify the individual and must be used in all research-related documents pertaining to that participant. This identification number must remain unchanged throughout the study; it should not be altered, including during re-screening of participants, after the initial assignment.

**Confirmation of TGW identity:** The study participants' transgender female identity is verified through a logical screening process using self-identified gender questions in the baseline questionnaire. Researchers rigorously assess the identity of the participants. If there are doubts about the identity of a participant, an additional identity confirmation questionnaire is sent, which includes questions about transgender-related knowledge. Additionally, the following methods are used to corroborate their identity: contacting the participant's recruiter to request identity verification evidence, reviewing the participant's social media posts for identity-related clues or evidence, and requesting the participant to provide recent photos of themselves presenting in feminine attire.

When the researchers determine that a participant meets the inclusion criteria, the investigator should record this decision and the date in the digital Case Report Form (CRF).

**7. STUDY PROCEDURES**

**7.1 Baseline**

Eligible participants will undergo an online informed consent process. After agreeing to participate in the study, participants will be invited to complete a questionnaire survey. The demographic and behavioural characteristics of the two groups of participants will be collected through an online survey platform (<https://jinshuju.net/home>).

**7.2 Follow Up**

For all participants, the study duration is six months from enrolment. All participants will receive follow-up surveys based on the online questionnaire platform at three and six months after enrolment.

**7.3 Intervention Contents**

**7.3.1 Quadruplex Self-Testing Package**

The quadruplex self-testing kits to be used (Wondlfo Guangzhou, China) are finger stick-based, which can detect antibodies of HIV-1, Treponema pallidum (TP), HBV, and HCV in blood specimens at 6 weeks after infection. The test kit will be packaged together with the necessary consumables (disposable retractable lancet, alcohol pad, capillary tube, Wondlfo solution, and a sealed bandage). Each package will include the corresponding identification number and instructions for the testing procedure, result interpretation, and result uploading. (Figure 2, 3).


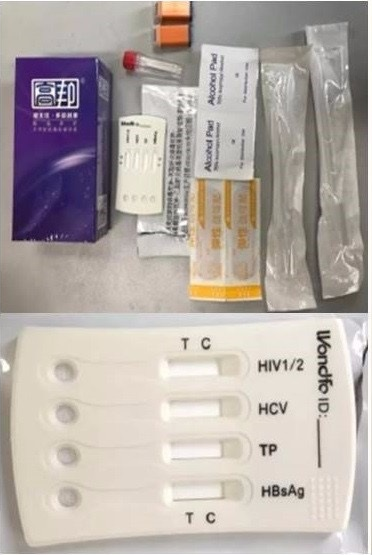


**Figure 2 HIV self-testing package details**


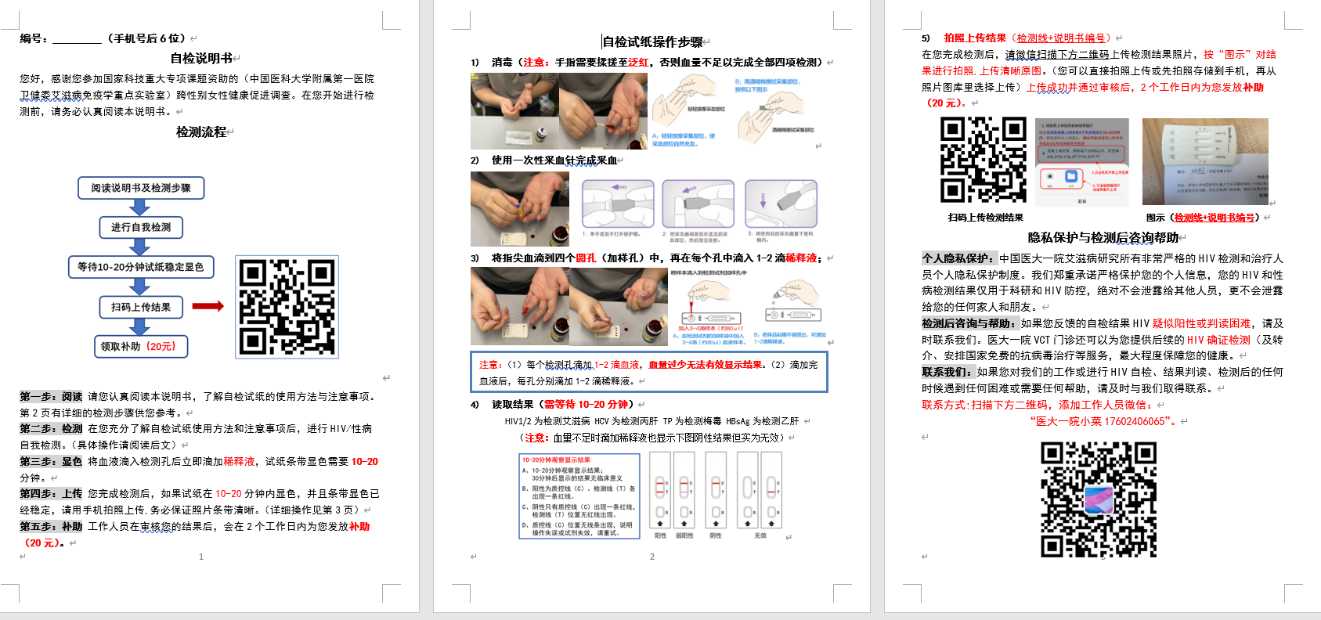


**Figure 3 HIV self-testing instructions**

**7.3.2 Online intervention**

HIV-related health information will be simultaneously sent to the intervention group and the control group via WeChat, with one message sent at enrolment and another message sent three months after enrolment. The content of the messages will include local testing locations for HIV (such as CDCs, hospitals, and CBO testing groups), information on HIV transmission routes, and prevention measures for HIV (such as condom use and PrEP).

**7.4 Intervention Procedures**

After including TGW who test negative for HIV and meet the eligibility criteria, they will be randomly assigned to the intervention group and the control group in a 1:1 ratio. Based on participants' needs, the intervention group will have the opportunity to apply for free HIV self-testing (HIVST) kits and mailing services every three months (via WeChat). Participants can request to receive 1-5 self-testing kits based on their own needs, and they are encouraged to share the HIVST kits with their partners. HIV-related health information will be regularly sent to both the intervention and control groups at predetermined intervals (at enrolment and three months after enrolment). Follow-up surveys will be conducted, and electronic questionnaires will be filled out at three months and six months after enrolment. To protect personal privacy, all HIV self-testing kits mailed to participants will be discreetly packaged in an unmarked box along with the instructions. Each self-testing kit will be assigned a unique code for future tracking and result retrieval. Users of the self-testing kits can scan the QR code on the instructions, take a photo of the self-test result, and anonymously upload it to the online platform (created through the online questionnaire platform: <https://jinshuju.net/home>).

**7.5 Measures of Different HIV Testing Results**

During the implementation of the study, researchers provide a series of counselling services to the participants before and after testing. They promptly review the result images uploaded by users of the self-testing kits. If the self-test result shows positive for HIV, the researchers will contact the individual immediately to provide appropriate counselling and referral services, including explaining the test result and referring them to medical institutions or Center for Disease Control and Prevention for HIV confirmatory testing, CD4 testing, and antiretroviral treatment services. If the HIV self-test result is negative, the participants will receive information about regular HIV testing and safe sexual behaviour.

**7.6 Questionnaire Contents**

The baseline questionnaire includes demographic characteristics (age, educational level, marital status, monthly income, etc.) and behavioural characteristics in the past three months (frequency of HIV self-testing kit use, frequency of HIV testing at venues, types and number of sexual partners in the past three months, condom use, drug use, inquiring about the HIV infection status of sexual partners, history of sexually transmitted diseases, age at first sexual intercourse, sexual orientation, etc.).

The follow-up surveys also include information about HIV testing results (whether disclosed to sexual partners), the number of HIVST kits shared with sexual partners, types of sexual partners, and knowledge of sexual partners' HIV infection status. Sexual partners who undergo HIV testing will be invited to complete a questionnaire, which includes demographic and behavioural characteristics, as well as the results of their most recent HIV test.

**8. WITHDRAWAL FROM THE STUDY**

**8.1 Decision to Withdraw from Participants or Investigators**

Participants have the right to withdraw from this study at any time for any reason without affecting their future rights to medical care and services at the institution. Participants may refuse to continue receiving the intervention at any time during the study, but can still remain involved in the research. Researchers may also decide to withdraw participants from the intervention measures, study procedures, or the entire study at any time before its completion.

**8.2 Reasons for Removal from Study**

Reasons for removal from the study include any of the following:

- Participants request
- Safety concern (such as concerns about being harmed by sexual partners)
- Withdrawal of consent from the study
- HIV seroconversion
- Loss of follow-up or death
- The decision by the investigator (If participants request test kits for purposes other than testing, such as obtaining HIV self-test kits for the purpose of selling them, rather than using them for their own and their sexual partners' HIV testing)

**9. ADVERSE EVENTS**

In the vast majority of cases, fingerstick blood collection conducted in this project is safe. However, in rare instances, some individuals may experience discomfort such as dizziness, and in extremely rare cases, they may experience blood donation reactions such as fainting. If participants experience any physical discomfort, they can contact our researchers at any time, and the medical personnel within the research team will provide timely guidance on post-blood collection reactions.

**10. STATISTICAL CONSIDERATIONS**

**10.1 Intention-to-Treat Set (ITT) Population**

The Intention-to-Treat Set (ITT) is defined as all randomized participants. The primary analysis population for all efficacy endpoints will be the Full Analysis Set (FAS) population.

**10.2 General Considerations**

Summary of participants' demographic and behavioural characteristics. Regarding continuous variables that follow a normal distribution, the mean and standard deviation will be used for description. In the case of variables that do not follow a normal distribution, the median and interquartile range will be used for description. For categorical variables, frequencies and percentages will be provided.

**10.3 Efficacy Analysis**

The following sections provide an overview of the planned analyses for the primary and secondary endpoints of the study's main intervention effects. All intervention effect analyses will be based on the intention-to-treat (ITT) population.

**10.3.1 Outcome Measure**

The primary outcome is the mean changes in the number of HIV tests among TGW during six months of follow-up.

The secondary outcomes include the mean changes in the number of HIV tests during three months, the proportion of HIV testing at six months, the proportion of HIV testing at three months, the mean changes in partner numbers during six months, and the mean changes in the frequency of condomless sex during six months.

We will rely on participants' reports to measure the HIV testing results. Completion of HIV testing is defined as sexual partners undergoing HIV testing based on venue or HIV self-testing (HIVST), uploading the HIVST results via the QR code in the test kit instructions, or directly reporting the HIVST results to the staff.

**10.3.2 Analysis of the Primary and Secondary** **Efficacies**

For the primary and secondary outcomes, covariance analysis will be used to compare differences in mean changes from baseline between groups, with group as a factor and baseline value as a covariate. A step-down approach will be used to find a significant result; the primary and secondary outcomes were tested sequentially to control the familywise type I error rate according to the following order: mean changes in the number of HIV tests at six months (primary outcome), mean changes in the number of HIV tests at three months, mean changes in the sexual partner number at six months, and mean changes in the frequency of condomless sex at six months. Statistical significance (two-sided P<0.05) at each step was required to test the next hypothesis. A linear mixed-effects model with individuals as a random effect will be used to evaluate the main effects of the intervention and interaction effects of time on the primary outcome.

**10.3.3 Other analysis of the primary intervention effect**

Subgroup analyses for the primary outcome will be performed based on age, education, occupation, income, marital status, and transient population. For sensitivity analysis, the results with imputation will be presented.

**10.4 Sample Size Considerations**

We used PASS V. 15.0 (NCSS, LLC. Kaysville, Utah, USA) to calculate the sample size based on a repeated measures design with two time points. Enrolment of 103 participants per group was projected to provided 90% power to detect a mean difference of 0.35 in the number of HIV tests per three months^1^ between groups (two-tailed α = 0.05). At least 115 patients per group were needed, assuming an anticipated 10% dropout rate.

**10.4.1 Tests for Two Means in a Repeated Measures Design in PASS**

Two-Sided Test. Null Hypothesis: D = 0. Alternative Hypothesis: D ≠ 0.

Covariance Type = Compound Symmetry

| Target Power | Actual Power* | N1 | N2 | N | **M** | **D1** | **Sigma** | Rho | Alpha |
| --- | --- | --- | --- | --- | --- | --- | --- | --- | --- |
| 0·90 | 0.90019 | 103 | 103 | 206 | 2 | 0.35 | 1 | 0.2 | 0·05 |

* Power was computed using the normal approximation method.

Note: Target Power is the desired power value (or values) entered in the procedure. Power is the probability of rejecting a false null hypothesis.

Actual Power is the power obtained in this scenario. Because N1 and N2 are discrete, this value is often (slightly) larger than the target power.

N1 and N2 are the number of items sampled from each population.

N is the total sample size, N1 + N2.

M is the number of time points (repeated measurements) at which each subject is observed.

D1 is the difference between the means of groups 1 and 2 under the alternative hypothesis.

Sigma is the standard deviation of a single observation. It is the same for both groups. Rho is the correlation between observations on the same subject.

Alpha is the probability of rejecting a true null hypothesis.

**10.4.2 Summary Statements**

Group sample sizes of 103 and 103 achieve 90% power to detect a difference of 0.35 in a design with 2 repeated measurements having a Compound Symmetry covariance structure when the standard deviation is 1, the correlation between observations on the same subject is 0.2, and the alpha level is 0.05.

**10.4.3 Parameter setting**

| Parameter | Setting |
| --- | --- |
| Alternative Hypothesis: | Two-Sided |
| Power: | 0.90 |
| Alpha: | 0.05 |
| Group Allocation: | Equal (N1 = N2) |
| D1 (Difference\|H1) | 0.35 |
| M (Number of Time Points) | 2 |
| Covariance Type: | Compound Symmetry |
| Sigma (Std Dev of a Single Observation) | 1 |
| Rho (Autocorrelation) | 0.2 |

**11. QUALITY CONTROL**

**11.1 Design Phase of the Protocol**

- Before the formal commencement of the study, a small-scale pre-survey will be conducted to refine personnel recruitment and inclusion, as well as improve the survey questionnaire.
- Training will be provided to the relevant staff members to ensure the quality of the research work.

**11.2 Implementation Phase of the Protocol**

- Strictly adhere to the inclusion criteria for survey participants and conduct rigorous screening and recruitment.
- Use a unique identification number to ensure the unique identity of recruited participants.
- Provide educational campaigns on HIV self-testing (HIVST) related knowledge to the TG individuals participating in the study. Ensure feedback rates and result authenticity by obtaining informed consent, providing reminders via phone or SMS, and offering a certain level of financial compensation.
- Minimize loss to follow-up during the survey process as much as possible.

**11.3 Analysis Phrase of Data**

Two survey personnel will be assigned to independently conduct data cleaning and data logic checks (e.g., range checks for data values). Any discrepancies will be resolved by third survey personnel.

**12. ETHICS**

**12.1 Informed Consent**

The investigator needs to provide a digital informed consent form. Before participants can take part in the study, the investigator must obtain the participant's informed consent in digital form after fully explaining the purpose, methods, anticipated benefits, and potential risks of the research. The participants should sign the digital informed consent form before any interventions take place. The digital informed consent form should be personally signed and dated by the participant. If a potential participant is illiterate or has impaired vision and does not have a legally acceptable representative, the investigator must provide an impartial witness to read the informed consent form to the participant and allow for questions. Subsequently, the participant and the witness must sign the informed consent form to demonstrate that the informed consent was given voluntarily and understood.

**12.2 Institutional Review Board/Independent Ethics Committee**

The protocol was developed prior to the start of the study. The plan was jointly discussed and signed by the researchers and the team leader, and it was submitted for approval to the Ethics Committee before implementation. If any issues arise during the implementation of this plan, the research protocol should be revised, and the revised protocol should also be submitted for approval to the Ethics Committee. Prior to the commencement of the study, researchers must provide participants with relevant details of the study, including the study content, objectives, potential benefits and risks, as well as the rights and obligations of the participants. During the study, all personal information of the participants will be kept strictly confidential.

**12.3 Confidentiality**

Restrict staff who have access to the personal information of participants and sign confidentiality agreements. Participants' personal information should be stored in locked cabinets at the workplace and supervised by designated personnel. CBO staff members should apply in advance for contact information before accessing participants' contact details, and use dedicated phones within the cabinets to communicate with participants. In all materials submitted to the project lead organization and the ethics committee, only the participants' and their partners' identification codes should be recorded.

The researchers unanimously agree that all information obtained from the project lead organization during the study is the property of the project lead, including manuals, agreements, and all other information related to the study. This information shall not be disclosed to any third party, except for individuals or institutions directly involved in this study or as required by law.

**13. DATA PROCESSING AND STORAGE**

**13.1 Database Establishment**

Statisticians should promptly establish a database, and after reviewing the database, investigators, statisticians, and research assistants will lock the data. To ensure data security, unauthorized personnel are not allowed to modify the data, and data must be backed up.

**13.2 Data Storage**

Investigators should ensure the integrity of the data. According to the principles of the Chinese “Guideline for Good Clinical Practice (GCP),” research data should be retained for at least five years.

**13.3 Data Availability**

All data from the study will be provided to the corresponding author, who has the ultimate authority to make decisions regarding the data.

**14. TIMELINE OF STUDY PLAN**

We plan to complete the initial participant recruitment and questionnaire design between June 2020 and September 2020. The preliminary survey and questionnaire revisions will be completed from October 2020 to December 2020. From January 2021 to December 2021, we aim to complete participant recruitment and a six-month follow-up. Data analysis is expected to be completed before March 2022.

**15. ROLE OF THE FUNDING SOURCE**

The study is to be funded by National Natural Science Foundation of China (82073620), and the Liaoning Revitalization Talents Program (XLYCYSZX1904). The funder of the study will have no role in the study design, data collection, analysis, interpretation, or writing of the manuscript.

**16. DECLARATION OF INTERESTS**

The principal investigators of the overall trial and each study site have no financial interests or other competing interests.

**17. REFERENCE**

1. Katz DA, Golden MR, Hughes JP, Farquhar C, Stekler JD. HIV Self-Testing Increases HIV Testing Frequency in High-Risk Men Who Have Sex With Men: A Randomized Controlled Trial. *Jaids-J Acq Imm Def* 2018; **78**(5): 505-12.

2. Poteat T, Wirtz AL, Radix A, et al. HIV risk and preventive interventions in transgender women sex workers. *Lancet* 2015; **385**(9964): 274-86.

3. Baral SD, Poteat T, Stromdahl S, Wirtz AL, Guadamuz TE, Beyrer C. Worldwide burden of HIV in transgender women: a systematic review and meta-analysis. *Lancet Infectious Diseases* 2013; **13**(3): 214-22.

4. Hibbert M, Wolton A, Crenna-Jennings W, et al. Experiences of stigma and discrimination in social and healthcare settings among trans people living with HIV in the UK. *Aids Care-Psychological and Socio-Medical Aspects of Aids/Hiv* 2018; **30**(7): 836-43.

5. Becasen JS, Denard CL, Mullins MM, Higa DH, Sipe TA. Estimating the Prevalence of HIV and Sexual Behaviors Among the US Transgender Population: A Systematic Review and Meta-Analysis, 2006-2017. *American Journal of Public Health* 2019; **109**(1): E1-E8.

6. Pitasi MA, Oraka E, Clark H, Town M, DiNenno EA. HIV Testing Among Transgender Women and Men-27 States and Guam, 2014-2015. *Mmwr-Morbid Mortal W* 2017; **66**(33): 883-7.

7. Best J, Tang WM, Zhang Y, et al. Sexual Behaviors and HIV/Syphilis Testing Among Transgender Individuals in China: Implications for Expanding HIV Testing Services. *Sexually Transmitted Diseases* 2015; **42**(5): 281-5.

8. Castro R, Ribeiro-Alves M, Correa RG, et al. The Men Who Have Sex with Men HIV Care Cascade in Rio de Janeiro, Brazil. *Plos One* 2016; **11**(6).

9. Chariyalertsak S, Kosachunhanan N, Saokhieo P, et al. HIV Incidence, Risk Factors, and Motivation for Biomedical Intervention among Gay, Bisexual Men, and Transgender Persons in Northern Thailand. *Plos One* 2011; **6**(9).

10. Oldenburg CE, Le B, Toan T, et al. HIV Pre-exposure Prophylaxis Indication and Readiness Among HIV-Uninfected Transgender Women in Ho Chi Minh City, Vietnam. *AIDS and behavior* 2016; **20**: S365-S70.

11. Figueroa C, Johnson C, Verster A, Baggaley R. Attitudes and Acceptability on HIV Self-testing Among Key Populations: A Literature Review. *AIDS and behavior* 2015; **19**(11): 1949-65.

12. Estem KS, Catania J, Klausner JD. HIV Self-Testing: a Review of Current Implementation and Fidelity. *Current HIV/AIDS reports* 2016; **13**(2): 107-15.

13. Tobin SC. WHO provides additional guidance on HIV self-testing and partner notification. *Aids* 2017; **31**(12): N17-n8.

14. Jamil MS, Prestage G, Fairley CK, et al. Effect of availability of HIV self-testing on HIV testing frequency in gay and bisexual men at high risk of infection (FORTH): a waiting-list randomised controlled trial. *The lancet HIV* 2017; **4**(6): e241-e50.

15. George G, Chetty T, Strauss M, et al. Costing analysis of an SMS-based intervention to promote HIV self-testing amongst truckers and sex workers in Kenya. *PLoS One* 2018; **13**(7): e0197305.

16. Ky-Zerbo O, Desclaux A, Boye S, et al. "I take it and give it to my partners who will give it to their partners": Secondary distribution of HIV self-tests by key populations in Côte d'Ivoire, Mali, and Senegal. *BMC Infect Dis* 2023; **22**(Suppl 1): 970.

17. Johnson CC, Kennedy C, Fonner V, et al. Examining the effects of HIV self-testing compared to standard HIV testing services: a systematic review and meta-analysis. *Journal of the International AIDS Society* 2017; **20**.

18. Pal K, Ngin C, Tuot S, et al. Acceptability Study on HIV Self-Testing among Transgender Women, Men who Have Sex with Men, and Female Entertainment Workers in Cambodia: A Qualitative Analysis. *Plos One* 2016; **11**(11).

19. Pant Pai N, Bhargava M, Joseph L, et al. Will an unsupervised self-testing strategy be feasible to operationalize in Canada? Results from a pilot study in students of a large canadian university. *AIDS research and treatment* 2014; **2014**: 747619.

20. Rosales-Statkus ME, Belza-Egozcue MJ, Fernández-Balbuena S, Hoyos J, Ruiz-García M, de la Fuente L. Who and how many of the potential users would be willing to pay the current or a lower price of the HIV self-test? The opinion of participants in a feasibility study of HIV self-testing in Spain. *Enfermedades infecciosas y microbiologia clinica* 2014; **32**(5): 302-5.

21. Pai NP, Sharma J, Shivkumar S, et al. Supervised and Unsupervised Self-Testing for HIV in High- and Low-Risk Populations: A Systematic Review. *Plos Medicine* 2013; **10**(4).

22. Truong HM, O'Keefe KJ, Pipkin S, et al. How are transgender women acquiring HIV? Insights from phylogenetic transmission clusters in San Francisco. *Aids* 2019; **33**(13): 2073-9.

23. Long JE, Ulrich A, White E, et al. Characterizing Men Who Have Sex with Transgender Women in Lima, Peru: Sexual Behavior and Partnership Profiles. *AIDS and behavior* 2020; **24**(3): 914-24.

24. Wirtz AL, Poteat T, Radix A, et al. American Cohort to Study HIV Acquisition Among Transgender Women in High-Risk Areas (The LITE Study): Protocol for a Multisite Prospective Cohort Study in the Eastern and Southern United States. *JMIR Res Protoc* 2019; **8**(10): e14704.

**Appendix A. Informed Consent**

**A randomized controlled study using HIV self-testing kits to promote HIV testing among transgender women and their sexual partners**

**Informed Consent**

This trial has been reviewed and approved by the Ethics Committee of the First Affiliated Hospital of China Medical University. We invite you to participate in the study entitled "A randomized controlled study using HIV self-testing kits to promote HIV testing among transgender women and their sexual partners". Participating in this project will benefit you by providing information about your own HIV and STI infection status. It will also give us an opportunity to understand the HIV and STI epidemic among the transgender community in our country and better provide health services for everyone. Before deciding whether to participate, please carefully read this informed consent form. This document provides detailed information about the research purpose, procedures, benefits to you, risks, discomforts, key study details, and your right to withdraw from the study at any time. If you have any questions regarding the informed consent form, please consult the research doctor responsible for this trial. If you decide to participate in this study, you will need to sign this informed consent form.

1. **Background**

HIV self-testing is a novel approach to HIV testing where individuals who want to know their HIV infection status can independently collect their own (blood/saliva) sample, perform the test, and interpret the results in a private setting. It effectively addresses concerns about personal privacy disclosure for the test takers. HIV self-testing offers advantages such as convenience, privacy, painlessness, and ease of use. The World Health Organization believes that this testing method can encourage more individuals who have not participated in HIV testing to get tested. HIV self-testing can also extend HIV testing to populations not covered by other HIV testing services. The transgender community, which faces a high risk of HIV infection, would benefit from this innovative testing method. HIV self-testing projects targeting the TG population have been implemented in several countries, such as the United States and Thailand, and have shown good acceptance and participation rates among local TG communities. Given the severe HIV epidemic in our country, particularly among the TG population with low HIV testing rates due to discrimination and other reasons, it is crucial to expand HIV self-testing services in China.

1. **Objectives**

This study aims to gather information on the HIV and STI infection rates, HIV testing practices of transgender (TG) participants, as well as their sexual partners, and related sexual behaviours through the distribution of HIV self-testing kits by mail and online questionnaire surveys. The study intends to provide reliable HIV epidemiological data for the country and offer a theoretical basis for implementing more scientifically grounded HIV prevention and control measures targeted at the TG population.

1. **Research process**

This project follows a 1:1 randomization principle and will be conducted over a period of six months. The intervention group will have the opportunity to apply for the intervention package immediately after enrolment. After HIV self-testing, participants from both groups are required to upload their test results by scanning a QR code. Additionally, both groups are required to complete a questionnaire every three months (with a subsidy of 50 yuan per questionnaire) until the end of the project. The specific inclusion criteria are as follows:

(1) Aged 18 to 65 years old.

(2) Assigned male at birth, and self-identifying as female.

(3) Tested negative for HIV antibodies during the screening at enrolment.

(4) Engaged in sexual activity in the past six months.

(5) Willing to provide truthful feedback on their sexual behaviour.

(6) Owns a smartphone and is able to use WeChat.

(7) Willing to sign an informed consent form.

(8) Subjects should fully understand the purpose, nature, and methods of the trial and still voluntarily participate in this trial and sign informed consent.

**4. Obligations**

To participate in this project, it is essential for you to be aware of and complete the following steps:

- You will need to complete online questionnaire surveys at baseline, three months, and six months. The research staff will notify you in advance through WeChat or other official digital platforms to complete the subsequent questionnaire surveys. The reminders will not include sensitive terms related to the HIV.
- If you are in the intervention group, you will need to provide necessary contact information (nickname/full name, contact phone number, address) so that we can mail the self-testing kits to you and get in touch with you if necessary.
- In order to ensure the accuracy of result interpretation and timely understanding of your HIV and sexually transmitted infection status, you need to scan the QR code inside the self-testing kit and upload a photo of your self-test result within 15-20 minutes after testing. We will assist you in interpreting the result. If your HIV self-test result is suspected positive, there is a possibility of false positives in a few individuals. Please contact us promptly, and we will provide you with free follow-up confirmatory testing and counselling services.
- The self-testing kit, disposable lancets, and pipettes are all single-use disinfectant tools. It is strictly prohibited to reuse or share them with others! Please handle the used self-testing kit and lancets properly by disposing of them in 84 disinfectant solution or soaking them in hypochlorous acid. Dispose of them in the medical waste container at a healthcare facility near your home.

**5. Potential benefits and risks**

In the vast majority of cases, finger-prick blood sampling is safe. However, some individuals may occasionally experience discomfort such as dizziness, and in extremely rare cases, they may experience a blood donation reaction, such as fainting. Medical personnel will provide timely guidance in case of any discomfort after blood sampling. Furthermore, this study does not involve any risks beyond routine medical procedures.

You will receive free HIV rapid screening, syphilis, hepatitis B, and hepatitis C rapid test kits. These tests are important for you to understand your infection status and guide appropriate treatment. You can also receive free condoms, lubricants, HIV awareness brochures, and professional HIV testing counselling. Additionally, you will receive a subsidy of 50 yuan after the baseline survey and each follow-up visit.

If your HIV rapid screening test is positive, we will provide you with further counselling and testing services. Your HIV test results will only be disclosed to you in person, and if necessary, we will arrange for you to receive free antiretroviral treatment provided by the government to ensure your health to the greatest extent possible.

If your HIV rapid screening test is positive, we will provide you with further counselling and testing services. Your HIV test results will only be disclosed to you in person, and if necessary, we will arrange for you to receive free antiretroviral treatment provided by the government to ensure your health to the greatest extent possible.

If you have any questions during the testing process or result interpretation, please seek assistance by leaving a message on the WeChat platform, sending an email, or calling the VCT Clinic hotline at the First Hospital of Medical University (detailed information will be provided to you by email).

**6. Participation and Withdrawal**

Participation in this study is voluntary. You have the right to refuse participation or withdraw from the study at any time. Your decision to do so will not result in discrimination, unfair treatment, or retaliation. Your medical care and rights will not be affected in any way.

**7. Confidentiality**

This study is an anonymous survey, and we solemnly promise to strictly protect your personal privacy. Your phone number and address information will only be used for sending self-testing kits to you. Your HIV and STI testing results will only be used for research and prevention purposes. They will never be disclosed to other individuals, including your family and friends. Please rest assured.

Furthermore, we want to emphasize that all the information related to your participation in this project will be kept strictly confidential. To ensure the privacy of the testers, the sender's information on the package, the external packaging, and payment records will not indicate any information related to HIV or testing kits.

**8. Opinions of participants**

I have read the informed consent form and have received relevant information about this project. I have had the opportunity to ask the researchers questions regarding the HIV self-testing project, and I have received answers to my inquiries.

I volunteered to participate in this study, and I am willing to cooperate with the medical staff and finish it carefully.

Signature: Date:

Phone number:

As the researcher, I have provided a thorough explanation and description to the participant regarding the purpose, procedures, potential risks, and potential benefits of this study. I have satisfactorily answered all the participant's relevant questions.

Signature: Date:

Phone number:

**Appendix B. Screening questionnaire**

S01. Your age is (in years). [fill in the blank]

S02. Your assigned sex at birth was？ (Single choice)

- Male
- Female

S03. How do you identify your current gender? (Single choice)

- Male
- Female
- Transgender female
- Intersex
- Uncertain

S04. Which of the following gender-affirming surgeries have you undergone? (Multiple selections allowed)

- No (Exclusive options)
- Breast augmentation
- Male genital and gonadal removal, female genital reconstruction
- Adam's apple reduction
- Facial feminization
- Other

**Appendix C. Online questionnaire (partly)**

Welcome to participate in the questionnaire survey for the Transgender Women's Health Promotion Project. This research project has been reviewed by the Ethics Committee of the First Affiliated Hospital of China Medical University. Your participation in this project will be beneficial for you to understand your own HIV and STI infection status, and it will also provide us with an opportunity to grasp the HIV and STI epidemic among the transgender community in our country, enabling us to better provide health services for everyone. This survey is anonymous, and we solemnly promise to strictly protect your personal privacy. We hope that you can truthfully complete the survey. Thank you sincerely for your participation!

**What is your PID? [fill in the blank]**

**Part A. Demographic Characteristics**

A1. What is your ethnicity? [Single choice]

- Han
- Manchu
- Tibetan
- Mongolian
- Uyghur
- Others

A2. What is your highest level of education? (Single choice)

- No formal education
- Primary school
- Junior high school
- Senior high school or vocational school
- Associate degree
- Bachelor's degree
- Master's degree or above

A3. What is your current occupation? (Single choice)

- Student
- Unemployed
- Employed with a stable job
- Sex worker/Entertainer
- Other: **________________________________________**

A4. What is your current average monthly income?

[fill in the blank] (Chinese Yuan)

A5. What is your current marital status? [Single choice]

- Single
- Cohabitation
- Living with a homosexual partner
- Living with a heterosexual partner
- Married
- Widowed

A6. What type(s) of medical insurance do you have? (Select one or more)

- None
- Employee Basic Medical Insurance
- New Rural Cooperative Medical Insurance
- Urban Resident Basic Medical Insurance
- Commercial Medical Insurance (purchased directly from insurance company)

A7. Who do you currently live with? (Single choice)

- Living alone
- Wife or female partner
- Male partner
- Parents/siblings/relatives
- Friends (roommates)/colleagues (co-renting)
- Dormitory (provided by school/workplace)
- Other

**Part B. HIV Testing Behavior**

B1. Have you been tested for the HIV in the past three months? (Single choice)

- Yes
- No

B2. How many times have you been tested for the HIV in the past three months? [fill in the blank]

B3. Have you participated in venue-based (VCT/hospital/CBO workgroup) HIV testing in the past three months? (Single choice)

- Yes
- No

B4. How many times have you been tested for venue-based HIV in the past three months? [fill in the blank]

B5. Have you used HIV self-testing (HIVST) for HIV testing in the past three months? (Single choice)

- Yes
- No

B6. How many times have you used HIVST for HIV testing in the past three months? [fill in the blank]

B7. Have you shared HIVST with your sexual partners in the past three months? (Single choice)

- Yes
- No

B8. In the past three months, which type of sexual partners have you shared HIVST with? (Single choice)

- Regular male sexual partner (i.e., boyfriend or male partner in a stable relationship for at least three months)
- Casual male sexual partner (i.e., non-commercial and non-transactional)
- Commercial male sexual partner (i.e., engaging in sexual activities with male partners in exchange for goods, money, or housing)
- Female sexual partner

B8. How many times have you shared HIV self-testing (HIVST) with your regular sexual partner in the past three months? [fill in the blank]

B9. How many times has your regular sexual partner been tested for HIV in the past three months? [fill in the blank]

B10. In the past three months, are you aware of the HIV infection status of your regular sexual partner? (Single choice)

- HIV negative
- HIV positive
- Not aware

B11. How many times have you shared HIVST with your casual sexual partner in the past three months? [fill in the blank]

B12. How many times has your casual sexual partner been tested for HIV in the past three months? [fill in the blank]

B13. In the past three months, are you aware of the HIV infection status of your casual sexual partner? (Single choice)

- HIV negative
- HIV positive
- Not aware

B14. How many times have you shared HIV self-testing (HIVST) with your commercial sexual partner in the past three months? [fill in the blank]

B15. How many times has your commercial sexual partner been tested for HIV in the past three months? [fill in the blank]

B16. In the past three months, are you aware of the HIV infection status of your commercial sexual partner? (Single choice)

- HIV negative
- HIV positive
- Not aware

**Part C. High-risk Sexual Behaviour**

C1. In the past three months, how many male sexual partners have you had anal sex with? [fill in the blank]

C2. When engaging in anal sex with male sexual partners in the past three months, how frequently did you use condoms? (Single choice)

- Always used
- Mostly used
- Occasionally used
- Never used
